# Nile red staining for rapid screening of plastic-suspect particles in edible seafood tissues

**DOI:** 10.1007/s00216-024-05296-8

**Published:** 2024-05-10

**Authors:** Julia Süssmann, Elke Kerstin Fischer, Lars Hildebrandt, Elke Walz, Ralf Greiner, Sascha Rohn, Jan Fritsche

**Affiliations:** 1 Department of Safety and Quality of Milk and Fish Products, Max Rubner-Institut, Federal Research Institute of Nutrition and Food, Hermann-Weigmann-Straße 1, 24103 Kiel, Germany; 2 Center for Earth System Research and Sustainability (CEN), University of Hamburg, Bundesstraße 55, 20146 Hamburg, Germany; 3 Department for Inorganic Environmental Chemistry, Helmholtz-Zentrum Hereon, Institute of Coastal Environmental Chemistry, Max-Planck-Straße 1, 21502 Geesthacht, Germany; 4 Department of Food Technology and Bioprocess Engineering, Max Rubner-Institut, Federal Research Institute of Nutrition and Food, Haid-Und-Neu-Straße 9, 76131 Karlsruhe, Germany; 5 Department of Food Chemistry and Analysis, Technische Universität Berlin, Institute of Food Technology and Food Chemistry, TIB 4/3-1, Gustav-Meyer-Allee 25, 13355 Berlin, Germany

**Keywords:** Microplastic quantification, Fluorescence microscopy, Nile red fluorescence, Image processing, Screening method

## Abstract

Concerns regarding microplastic (MP) contamination in aquatic ecosystems and its impact on seafood require a better understanding of human dietary MP exposure including extensive monitoring. While conventional techniques for MP analysis like infrared or Raman microspectroscopy provide detailed particle information, they are limited by low sample throughput, particularly when dealing with high particle numbers in seafood due to matrix-related residues. Consequently, more rapid techniques need to be developed to meet the requirements of large-scale monitoring. This study focused on semi-automated fluorescence imaging analysis after Nile red staining for rapid MP screening in seafood. By implementing RGB-based fluorescence threshold values, the need for high operator expertise to prevent misclassification was addressed. Food-relevant MP was identified with over 95% probability and differentiated from natural polymers with a 1% error rate. Comparison with laser direct infrared imaging (LDIR), a state-of-the-art method for rapid MP analysis, showed similar particle counts, indicating plausible results. However, highly variable recovery rates attributed to inhomogeneous particle spiking experiments highlight the need for future development of certified reference material including sample preparation. The proposed method demonstrated suitability of high throughput analysis for seafood samples, requiring 0.02–0.06 h/cm^2^ filter surface compared to 4.5–14.7 h/cm with LDIR analysis. Overall, the method holds promise as a screening tool for more accurate yet resource-intensive MP analysis methods such as spectroscopic or thermoanalytical techniques.

**Graphical Abstract:**

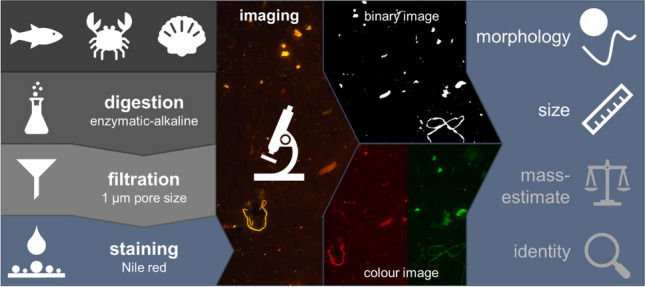

**Supplementary Information:**

The online version contains supplementary material available at 10.1007/s00216-024-05296-8.

## Introduction

Microplastic (MP) particle contamination is a pervasive and concerning environmental issue that gained considerable attention in recent years due to its potential impact on aquatic ecosystems and human health. These synthetic polymer particles are characterised by a size range of 1–5000 µm and water insolubility [1]. MP originate from the release of small-scale plastics, mechanical wear of plastic products like textile fibre release, or degradation of plastic waste in the environment [2]. With plastics being predominant among marine debris [3], aquatic organisms are susceptible to MP exposure via adherence or ingestion [4, 5]. Reports on MP presence in commercial seafood species and edible tissues [6] highlighted a possible path for plastic particles entering the food chain with processing and packaging contributing even further to an entry point for MP [7–9]. Recent findings of MP in human body fluids and tissues [10] emphasise the need for monitoring MP occurrence in food, including seafood, for a better understanding of the role of human dietary MP exposure.

A major challenge of monitoring MP is the complex nature of the analyte(s), with a great variety in size, chemical composition, and presence of certain additives. MP may undergo further modifications when exposed to environmental processes by ageing, change of surface charge, or formation of a corona of additional substances (e.g. proteins) [11]. The complex matrix of seafood poses another challenge, requiring thorough sample preparation to minimise matrix residues potentially interfering with MP detection [11, 12]. Infrared or Raman microspectroscopy (µ-FTIR, µ-Raman) is among the most well-developed and widely used methods for particle-based MP analysis, reducing false-positive findings by chemical identification of particles [12]. Therefore, their application was recommended by recent standardisation actions [1, 13], as well as for regulatory use, e.g. by *California’s State Water Resources Control Board* [14]. Due to their long analysis times of up to several hours or even days per sample, the suitability of µ-FTIR or µ-Raman for routine analysis, monitoring purposes, and regulatory purposes is still limited [14]. While state-of-the-art techniques like laser direct infrared (LDIR) imaging demonstrated higher potential for time-efficient MP analysis of water samples [15], data on performance for matrix-rich samples like seafood is limited. Commonly used mass-based approaches, like pyrolysis gas chromatography–mass spectroscopy (Py-GC/MS), enable higher sample throughput, while offering quantitative information on MP [16]. These methods do not provide information on particle sizes though, and signals of a few larger particles can mask the presence of smaller ones due to an exponential relation of particle size and mass [11].

In recent years, detecting MP with fluorescence microscopy after labelling with a fluorophore tag, most prominently Nile red (NR), has gained increasing popularity [11, 12]. With a resolution in the lower micron range, fluorescence microscopy of NR-stained particles allowed for highly sensitive MP determination [17]. Especially when combined with (semi-)automated analysis, the method showed great potential as a cost- and time-effective tool for MP analysis [18, 19]. Further potential for nanoplastic analysis of NR-stained samples is indicated when coupled with single particle tracking or flow cytometry [20, 21]. NR is a lipophilic and photostable dye with a strong affinity for non-polar materials, and has already been used for early analyses of MP [22–24]. However, NR also interacts with other organic components like lipids or proteins [23, 25], both occurring in seafood with significant amounts. Residual sample material such as fats, soaps, or gels can hinder the identification of fluorescent MP and increase the risk of false-positive MP detection [26, 27]. Particle counting without any further differentiation of fluorescent particles can consequently result in a severe overestimation of MP in biota samples [28, 29]. As NR fluorescence is sensitive to the chemical polarity of its surroundings [24], MP and particles of natural origin (PNO) can potentially be at least partially differentiated by colour and brightness [27]. Accurate analysis, however, strongly relies on operator experience and needs extensive training [27, 30, 31]. Furthermore, metadata or threshold limits for differentiating MP from PNO are often not provided, thus, limiting the comparability of results [27]. Maes et al. [32] proposed a mathematical approach for differentiating MP and natural residues by colour and digital image analysis, and reducing operator bias. Meyers et al. [18] recently demonstrated that the application of machine learning for automated identification of NR-stained particles achieved fast and accurate MP detection in environmental samples (differentiation of MP and PNO for ≥ 93% of particles spiked to mussels; correct polymer type assignment for 80% of particles) [18].

Screening methods are needed to overcome research restraints on MP occurrence in food, especially in seafood. While the potential of fluorescence microscopy after NR staining for cost- and time-efficient MP detection in various environmental samples was demonstrated in recent years, its potential for MP analysis in seafood with high protein or lipid contents that potentially interfere with MP detection has yet to be explored. The present study aimed at developing a rapid method for detecting MP in seafood. Commercially relevant seafood samples were therefore stained with NR after enzymatic-alkaline digestion and membrane filtration. Semi-automated data processing was tested for reducing operator bias. Staining and measurement parameters were optimised for seafood and threshold values for differentiating MP and matrix-inherent PNO by fluorescence were established. Method performance was exemplarily assessed with spiked fish fillet. Sample throughput and plausibility of results were assessed by comparing the proposed method with LDIR imaging, a state-of-the-art technique for MP detection in environmental samples.

## Material and methods

### Reagents and material

Liquid pepsin (660 u Ph. Eur./mL) was obtained from AppliChem GmbH (Darmstadt, Germany). Evans blue dye, NR dye, and n-hexane (C_6_H_14_) were obtained from Carl Roth GmbH & Co. KG (Karlsruhe, Germany). Carbon disulphide (CS_2_) and Tween20® were obtained from Honeywell International Inc. (Wabash, IN, USA). Calcofluor white staining agent, chloroform (CHCl_3_), dichloromethane (CH_2_Cl_2_, DCM), fuming hydrochloric acid (HCl), and potassium iodide (KI) were obtained from Merck KGaA (Darmstadt, Germany). Acetone (C_3_H_6_O), ethanol (C_2_H_6_O), hydrogen peroxide (H_2_O_2_), isopropanol (C_3_H_8_O), and potassium hydroxide (KOH) were obtained from Th. Geyer & Co. KG (Renningen, Germany). All chemicals were of analytical purity grade.

#### Reference particles of synthetic and natural polymers

Commercially relevant plastic particles and nurdles [33], UV-aged MP, coloured household MP, potential procedural contaminants (cotton fibres), and PNO potentially occurring in edible tissues of seafood were selected for establishing fluorescence threshold values for MP identification of NR-stained particles. Synthetic polymers were provided by the Bundesanstalt für Materialforschung und -prüfung (BAM, Berlin, Germany), referred to as BAM-MP. Further polymers were purchased from Goodfellow Cambridge Ltd. (Lille, France), and Alfa Aesar (Haverhill, MA, USA), referred to as in-house reference. PNO were obtained from fishbone, shrimp shells, mussel shells, and cotton. When necessary, small particles (≤ 500 µm) were obtained by cutting, precipitation, or ultra-centrifugal milling and consecutive sieving with stainless-steel sieves. A comprehensive list of materials is provided in the supplementary information (SI), Table S1. For sample spiking, the most commonly detected MP in food were selected, namely nylon 6 (PA6), polyethylene (PE), polyethylene terephthalate (PET), polypropylene (PP), polystyrene (PS), and polyvinylchloride (PVC) [34]. Furthermore, nylon 12 (PA12) was sieved with a 50 µm and 25 µm stainless-steel mesh for spiking with particles within a small size range. Particles were suspended in solutions of Tween20® and KI or isopropanol depending on the polymer density (Table 1). Particle counts of each spiking suspension were determined by pipetting 100 µL-aliquots (*n* = 5) onto glass fibre filters, consecutive NR staining, fluorescence microscopy, and image analysis as described in “Sample analysis”. For pipetting, a displacement pipette (Transferpettor™, Brand GmbH & Co. KG, Wertheim, Germany) equipped with a glass capillary (1.95 mm opening) was used. To avoid particle sedimentation or floatation, the particle suspension was shaken vigorously before each pipetting step.

**Table 1:** Composition of MP suspensions used for sample spiking; detergent–aqueous 0.5% Tween20®-solution; *RSD* relative standard deviation; *rcv* recovery of polymer mass estimate based on weighed particle mass

Polymer	Dominant size (95% of particles)	Particle count (MP/mL)	RSD (%)	Mass estimate (µg/mL)	Suspension solution
PA6	10–50 µm	1287 ± 387	30	69 ± 53 (rcv = 111%)	0.9 mol/L KI in detergent
PE	10–450 µm	3210 ± 957	30	63,463 ± 25,783 (rcv = 105%)	Ethanol:detergent (55:45, *v/v*)
PET	10–100 µm	2613 ± 325	12	359 ± 57 (rcv = 82%)	4.0 mol/L KI in in detergent
PP	10–500 µm	358 ± 71	20	215 ± 83 (rcv = 62%)	ethanol:in detergent (50:50, *v/v*)
PS	10–60 µm	941 ± 321	34	322 ± 137 (rcv = 75%)	0.5 mol/L KI in detergent
PVC	20–350 µm	836 ± 138	17	7562 ± 2850 (rcv = 92%)	5.0 mol/L KI in in detergent
PA12	25–50 µm (rounded shape)	478 ± 295	62	-	detergent

### Prevention of procedural contamination

Experiments were conducted in a laboratory with restricted access wearing a white cotton lab coat and trousers. Filtration and filter treatment (oxidation, staining) took place within a laminar flow box. All liquids (reagents, solvents, water) were filtered with glass fibre filters (0.7 µm particle retention, Th. Geyer & Co. KG, Renningen, Germany) directly before use. Labware not suited for thermal cleaning (e.g. filtration apparatus, PTFE-coated stirring rods, heat-sensitive filter membranes) was rinsed three times with 10 mL deionised water (DI water, generated with a reverse osmosis system and additional mixed bed filter). Glass slides and flasks (covered with aluminium foil) and glass fibre filters (stored in Petri dishes) were heated at 500 °C for 5 h. Glass flasks were additionally rinsed with 10 mL DI water prior to use. Preliminary analysis of singular potential contamination sources (e.g. glassware, deposition from air, reagents) indicated a high randomness of each individual source. Therefore, three procedural blank samples were prepared for each sample series to account for the total contamination of the respective series. The procedural blanks were prepared and analysed like matrix samples but using the respective amount of DI water instead of seafood.

### Sample preparation

#### Homogenisation and spiking of seafood matrix

Whole herring (*Clupea harengus*), and fresh salmon fillets with skin (*Salmo salar*) were purchased from a local market and transported on ice in an expanded polystyrene box as supplied by the merchant. Frozen whitefish fillet (*Theragra chalcogramma*), shrimps (*Penaeus longirostris*), and fresh mussels (*Mytilus edulis*) were purchased pre-packaged from German retail stores. Non-edible tissues (skin, shells, fishbone, innards) were removed. Fish fillets, shrimp tails, and mussels’ tissues were then homogenised with a commercial stainless-steel hand blender. Samples were stored at − 20 °C in aluminium cups covered with aluminium foil.

A subset of samples was spiked with MP for recovery tests and method comparison. MP mass estimation was evaluated with weighed BAM-MP spiked to 1–2 kg aliquots homogenised herring fillet in different concentrations (0.75 mg/kg, 30.18 mg/kg, and 234.66 mg/kg). The spiked homogenates were mixed again with a hand blender. Subsequently, 1 g and 10 g aliquots were weighed into glass flasks (*n* = 5). Particle counting was evaluated by spiking pre-homogenised salmon fillet with 100 µL in-house reference MP suspensions (Table 1) in a mixture (PA6, PE, PET, PP, PS, PVC) and with PA12 (*n* = 5 each). Additionally, 10 mL filtered DI water was spiked with the same amount of each spiking suspension (or only PA12) and immediately filtered (*n* = 3).

#### Extraction of MP from edible seafood tissue

Aliquots of 10 g homogenised seafood were digested with a two-step procedure as described by Süssmann et al. [35]. First, the sample was digested with 90 mL of a 1% pepsin solution in 0.063 mol/L HCl (stirring for 2 h at 40 °C). Afterwards, 10 mL 50% KOH solution (50:50, *w/w* in water) was added (stirring for 4 h at 40 °C) [36]. MP was isolated from most digested samples by vacuum filtration using Ø 47 mm PTFE filters (pore size 1–2 µm; Pieper Filter GmbH, Bad Zwischenahn, Germany). Spiked herring fillet was filtered with silver filters (pore size 0.8 µm; Pieper Filter GmbH, Bad Zwischenahn, Germany). PA12-spiked salmon was filtered with glass fibre filters (particle retention 1.2 µm; Th. Geyer GmbH & Co. KG, Renningen, Germany). The glass flask and filtration apparatus were rinsed three times with 10 mL DI water and once with 10 mL isopropanol. The filters were then placed in glass Petri dishes, covered with 2 mL H_2_O_2_ solution (15% in DI water, *v/v*) and dried for 48 h at room temperature.

#### Staining of sample filters

Optimal conditions for NR staining of edible seafood samples were determined with preliminary tests (SI section 2.5). Seafood samples were stained with 1 mL NR in hexane (*c* = 50 µg/mL) for 30 min at 40 °C. Afterwards, 1 mL NR in ethanol:acetone (1:1, *v/v*; EtAc; *c* = 50 µg/µL) was added to the filters and incubation was repeated for 30 min at 40 °C. After cooling to room temperature, excess dye was removed from the filter surface by rinsing with 5–10 mL isopropanol.

A subset of seafood samples was additionally stained with 0.5–1 mL Calcofluor white staining (10 min incubation at room temperature) after optimised NR staining [36] in order to test the effects of counterstaining on MP detection in seafood.

### Sample analysis

#### Analysis with fluorescence microscopy and semi-automated image processing

Particles on the filter were detected and analysed by fluorescence microscopy using the Axioscope 7 equipped with an Axiocam 503 colour camera (both Carl Zeiss AG, Germany), 565 nm LED illumination (5% intensity, 100 ms exposure), and an orange filter. Samples were observed with a 2.5 × or 5 × objective (10 µm or 5 µm resolution respectively). No colour correction was applied. A subset of samples was analysed with a 10 × objective (1 µm resolution). Due to the 10 × objective’s low depth of field, a *z*-stack of the sample was measured and a 2D image was generated by applying maximum projection.

Particle size, morphology, brightness, and colour were obtained with image analysis. Therefore, binary images of the scans were generated with Adobe Photoshop® (manual adjustment of brightness and contrast, separation of particles and background). Morphological attributes were obtained by analysing binary images with ImageJ. Isolated pixels (artefacts from image editing) were removed using the “open” function. Fluorescence colour and total particle brightness (TPB) were obtained with the original colour images.

#### MP estimation and procedural blank correction

Particle numbers were assessed in size classes of 5–10 µm, 10–50 µm, 50–100 µm, 100–500 µm, 500–1000 µm, and 1000–5000 µm [16]. Each size class was further separated by morphology and fluorescence. For particle mass estimation, the two-dimensional particle morphologies were approximated to three-dimensional objects, namely spheres (spheroids), cuboids (fragments,) and cylinders (fibres) for volume calculation. Details on the calculations are provided in SI section 2.2. The number and mass of MP suspect particles of each series were corrected by the respective procedural blanks [37]. Therefore, a limit of quantification (LOQ) was calculated based on the mean particle number of the respective procedural blanks plus ten times the standard deviation for each combination of size class, morphology, and fluorescence group. Results exceeding the LOQ were corrected by subtracting the mean particle number or mass estimate of the procedural blanks for the respective particle type or mass category.

#### Quantum cascade laser-based laser direct imaging analysis

The proposed method was compared with LDIR imaging for assessing the plausibility of MP analysis. Therefore, MP-spiked samples were first analysed with fluorescence microscopy and then with LDIR imaging (8700 LDIR Chemical Imaging System, Agilent Technologies Inc., Santa Clara, USA).

Aliquots of 1 g (*n* = 7) of spiked herring fillet homogenate were digested with 9 mL pepsin solution and 1 mL KOH. Five samples were filtered with glass fibre filters (1.2 µm particle retention, 25 mm diameter) from Th. Geyer & Co. KG (Renningen, Germany). Two samples were filtered with polyethylene terephthalate glycol (PETG) gold-coated membrane filters (0.2 µm pore size, 100/0 nm coating, 25 mm diameter) from Sterlitech Corp. (Auburn, WA, USA), as required for LDIR analysis. Due to the small pore size (0.2 µm), the filters clogged rapidly and filtration was aborted after 20 min, discarding the remaining liquid. The filters were rinsed once with 3 mL filtered DI water and placed onto GFF stored in Petri dishes for better soaking with H_2_O_2_. After drying, the samples were stained with NR (c.f. 2.3.3). The stained filters were mounted on specialised filter holders (Agilent Technologies Inc., Santa Clara, USA) and imaged with fluorescence microscopy (c.f. 2.4.1). The sample holder was then stored in a Petri dish for transport and analysed with LDIR imaging [15, 38]. Hereby, the size fractions 10–100 µm and 100–5000 µm were analysed separately using the Clarity Software (Version 1.5.58, Agilent Technologies Inc., Santa Clara, USA). Accordingly, the datasets were merged and evaluated applying a hit quality index of 0.85 (Pearson’s correlation coefficient of 1st derivatives of IR spectra) by means of a custom-written Excel^©^ spreadsheet.

## Results

### MP detection and differentiation from PNO with optimised staining conditions

#### Impact of sample preparation on polymer integrity and fluorescence

**Fig. 1:**
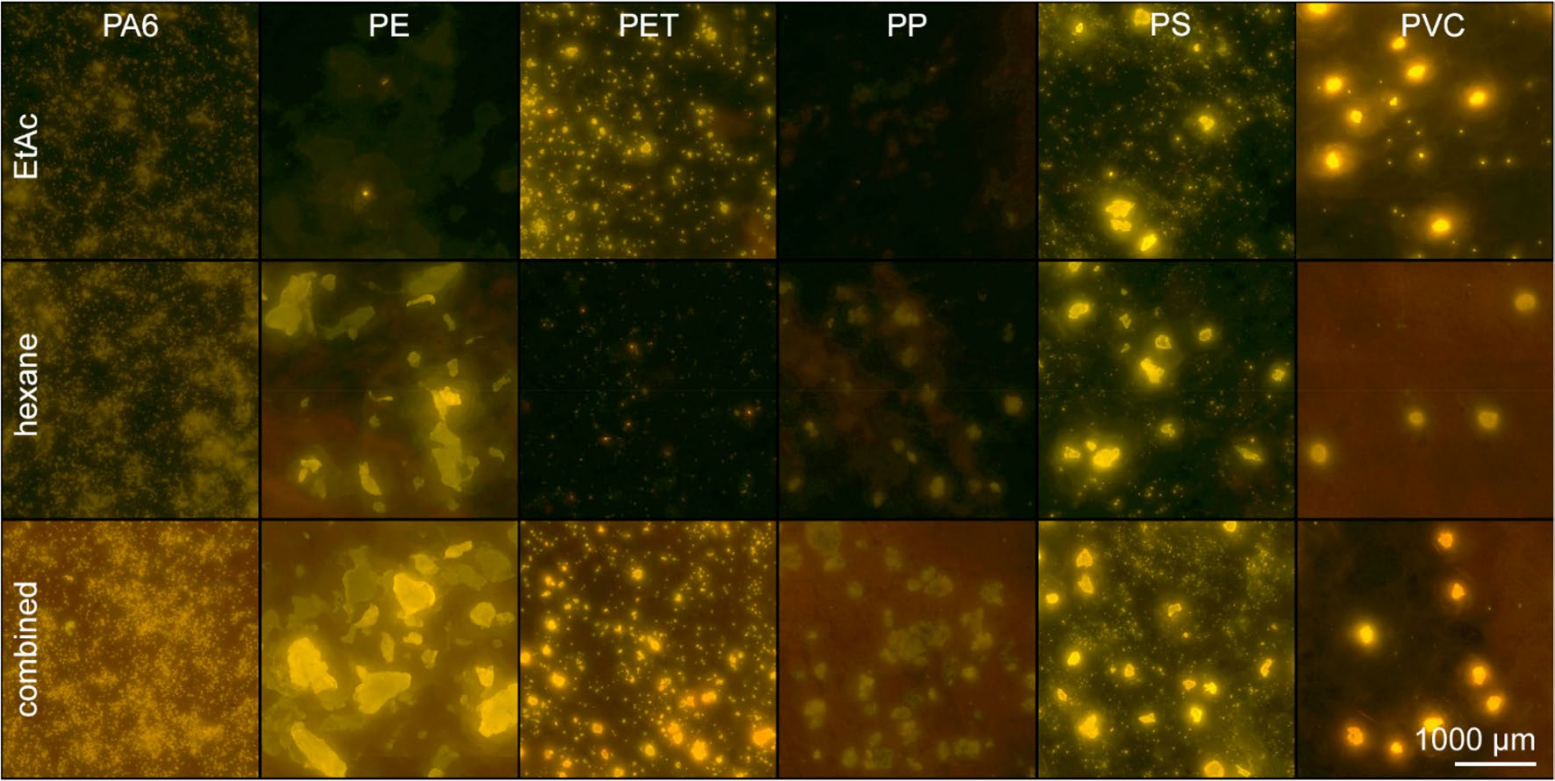
Photographs of in-house reference MP stained two times with 100 µL of a 100 µg/mL NR solution in EtAc and hexane each. For the combined approach, samples were stained once with 100 µL of each solution. Samples were observed with fluorescence microscopy. The brightness was adjusted digitally to increase the visibility of weakly stained MP (PE, PET, PP). The unedited image is provided in the SI (Fig. S13)

The digestion method had a negligible impact on polymer integrity [35], particle brightness, and fluorescence colour (Fig. S5). However, MP staining could affect polymer integrity by MP dissolution. NR dissolved in alcohols (e.g. isopropanol) had the least effect on MP dissolution but also stained MP most weakly (Fig. S12). Brightest staining was achieved with NR dissolved in hexane or EtAc, depending on the polymer type, as shown in Fig. 1. For staining MP mixtures of different chemical polarity (e.g. PE and PET), a two-step staining procedure was therefore preferred.

**Fig. 2:**
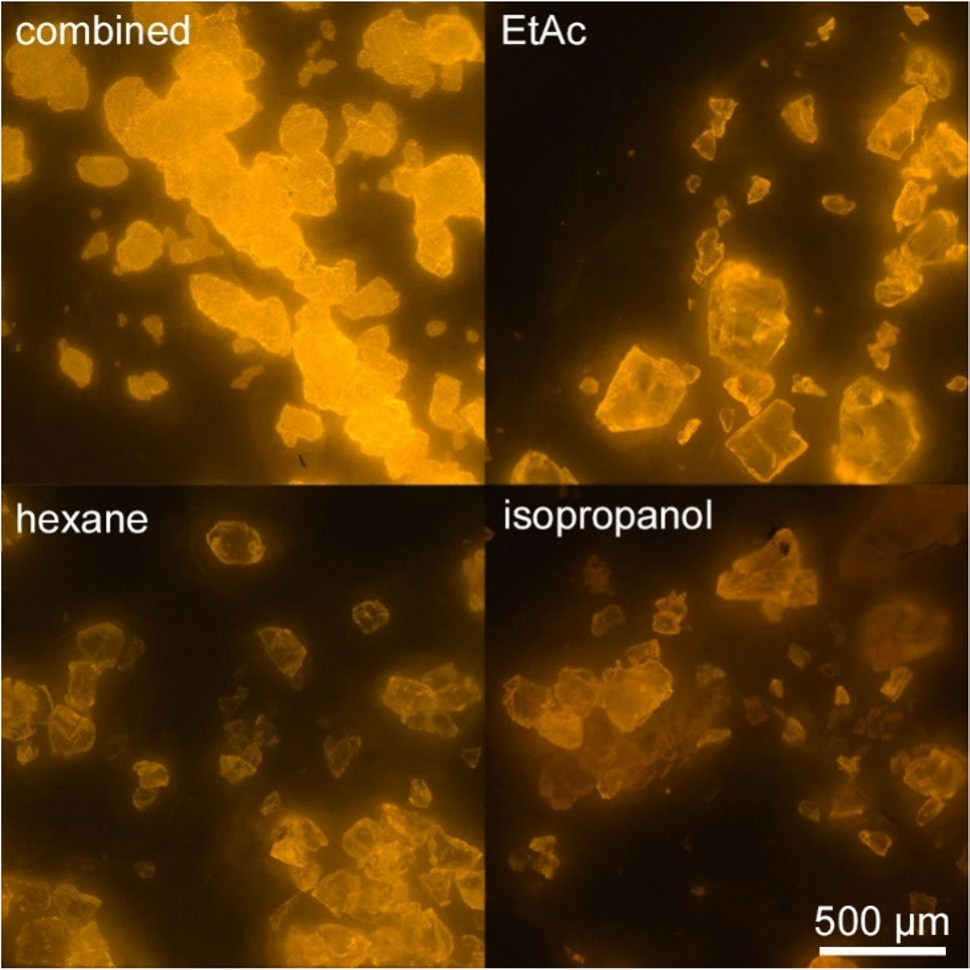
Photographs of BAM-PS on glass fibre filters, observed with fluorescence microscopy (*λ*_ex_ = 565 nm). Particles (*n* > 1000 each) were stained with NR dissolved in different solvents by moistening the filter and incubation for 30 min at 40 °C per solvent

**Fig. 3:**
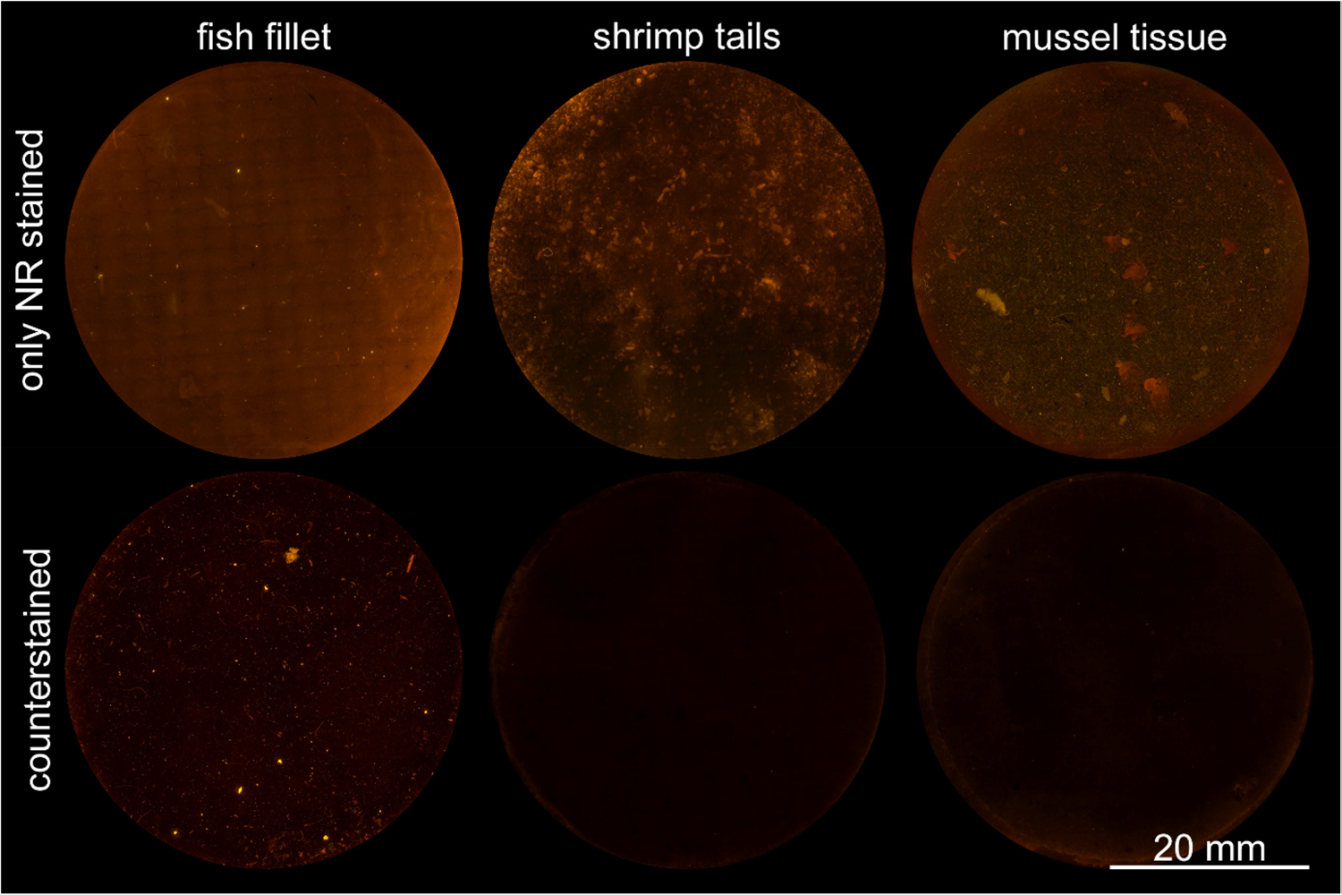
Photographs of digested seafood samples (edible tissues of whitefish, shrimps, mussels) on glass fibre filters, observed with fluorescence microscopy (*λ*_ex_ = 565 nm). Different aliquots of the same homogenate were analysed after staining with 100 µg/mL Nile red in EtAc alone (upper row) and after additional staining with Calcofluor white (lower row)

Of the six most relevant polymers, PS was the most sensitive to swelling and dissolution. A preliminary test indicated that especially expanded PS was rapidly dissolved by acetone, or DCM and shrank in contact with hexane as illustrated in Fig. 3. Therefore, partial dissolution of PS during staining could not be excluded. For the preliminary staining tests, MP was submerged in solvents. To mimic seafood staining conditions, BAM-PS (solvent-sensitive) was additionally stained with NR solutions on glass fibre filters. Reduced particle opacity and roundness of BAM-PS stained with NR dissolved in EtAc or the combined approach indicated partial dissolution (Fig. 2). Image analysis revealed a loss of particles smaller 50 µm of 17% (hexane), 28% (EtAC), and 55% (combined solvents) compared to staining with isopropanol. Simultaneously, the number of large particles (≥ 50 µm) increased to 42%, 70%, and 140% respectively. Particle size distributions of stained BAM-PS are illustrated in Fig. S15. Particle brightness correlated negatively with the effects of partial polymer dissolution. Brightest staining of particles was achieved with the combined approach with a TPB of 88 ± 18, followed by EtAc (TPB = 68 ± 19), hexane (TPB = 52 ± 15), and isopropanol (TPB = 26 ± 14).

#### Improved differentiation of MP and PNO

When staining with NR dissolved in isopropanol (least impact on polymer morphology), a mean of 88 ± 16% of particles were correctly classified as MP (ß-error of 12 ± 16%, Table S4). This was mainly attributed to the high ß-error of 45% for PE detection (Table S4). The optimised method improved especially the staining of PE, resulting in ß-errors for MP suspect classification of 4% or less (Table S4). With the optimised staining method, 1% of chitin particles were incorrectly classified as MP (false-positive classification, α-error). Other seafood-related PNO were not classified as MP. Counterstaining overall reduced MP fluorescence, but not to a significant extent (Fig. S6). Classification of MP from other sources was less reliable, e.g. indicated by the analysis of BAM-MP with ß-errors of up to 100% (Table S4, BAM-PVC).

The identification of polymer type was not feasible due to similar fluorescence. When staining with NR dissolved in isopropanol, mean ß-error rates of 27 ± 20% were achieved (1 *SD*, *n* = 6; Table S5). Due to the overall increased fluorescence of MP when staining with the optimised method, the differences between polymers were even lower, resulting in mean ß-error rates of 62 ± 27% (1 *SD*, *n* = 6). Therefore, only total MP occurrence was evaluated for the spiking experiments.

The residual tissue of digested seafood (whitefish fillets, shrimps, mussels) was stained deep blue with counterstaining as described by Helmberger et al. [36]. This resulted in reduced background fluorescence as can be seen in Fig. 3, beneficial for particle recognition and binary image generation. Additionally, the blue stain of tissue residues improved the differentiation of MP and PNO with light microscopy, for example when selecting particles for a consecutive follow-up µ-Raman analysis (data not shown). As the background fluorescence of fish fillet was lower compared to mussels or shrimps, the effect of counterstaining was less pronounced (Fig. 3, first column).

#### Limitations of detection and identification by MP inherent properties

Intrinsic particle properties (e.g. morphology, colour, ageing) potentially affect MP detection after NR staining [39]. Therefore, limitations of MP detection were exemplarily explored with household plastics and artificially UV-aged MP, presented in detail in SI section 2.4. Inherent particle colour considerably affected fluorescence (Fig. S8), for example preventing the detection of black MP due to weak fluorescence. Fibres were detected to a lesser extent compared to particles (e.g. detection rate of polyester fibres decreased by 90% compared to the PET in-house reference). This was attributed to their smooth surface, which was supported when comparing the fluorescence of rough and smooth PP particles generated from the same bottle cap (TPB = 45 ± 4 and 49 ± 2 respectively, Fig. S7). Artificial UV aging also influenced polymer fluorescence, resulting in diminished TPB (Table S6). Red fluorescence (influenced by chemical polarity) either increased (PE, PP) or decreased (PA6). Detection rates of UV-aged MP consequently decreased to 84–0%.

### Plastic recovery in spiked fish fillets

#### Procedural contamination during sample preparation

All procedural blanks were contaminated with MP suspect objects. The least contamination occurred during the preparation of 1 g samples with 1–6 MP/sample (≤ 0.4 µg), followed by the PA12 series with 50–79 MP/sample (0.1–0.3 µg). Twenty-six to 832 MP/sample (0.44–21 µg) were detected in blanks of the BAM-MP series, and 912–1627 MP/sample (2–124 µg) in blanks of the MP mixture series. Most contamination were rounded particles of medium fluorescence. Mean particle numbers and mass estimates of procedural blanks as well as LOQ values used for blank correction (c.f. 2.4.2) are listed in Tables S8 and S9. The lower contamination of the 1 g sample series was attributed to lesser reagent volume for digestion (100 mL → 10 mL), smaller glass flasks and filtration funnels (250 mL → 15 mL), and smaller filter area (17.3 cm^2 ^→ 4.9 cm^2^. Samples of the PA12 series were filtered with glass fibre filters (cleaned by heating) and were not fixed between glass slides, as opposed to the MP mixture and BAM-MP series. Contamination might therefore stem from insufficiently cleaned membrane filters or glassware. With contamination of filter membranes being a common source of procedural contamination, glass fibre filters are often recommended for MP isolation as they can be effectively cleaned by thermal treatment [40].

#### Recovery of MP counts and mass estimates in spiked samples

Due to the high variances, results are not presented with average values but the total range of results and relative standard deviation (RSD) between samples, instead.

The estimated MP mass in the native herring fillet was below the LOQ (0.5–4.2 MP/g, RSD = 109%). Recoveries of MP mass estimates in spiked herring ranged from 16 to 37% (RSD = 41%) at 0.75 mg/kg spiking level, 8–19% (RSD = 28%) at 30 mg/kg, 3–37% (RSD = 64%) at 235 mg/kg (10 g aliquot), and 7–30% (RSD = 33%) at 235 mg/kg (1 g aliquot). Corresponding MP counts varied greatly with an RSD of 143%, 181%, 46%, and 76% respectively. The low recovery of BAM-MP was attributed to the weaker fluorescence compared to in-house reference MP (c.f. 3.2.1; Table S4). No correlation of MP counts and mass estimates was observed due to the inhomogeneous particle size distribution between aliquots (90% of particles ≤ 50 µm at spiking level 0.75 mg/kg; 60–70% of particles ≤ 50 µm at the other spiking levels). This might be caused by inhomogeneity when weighing BAM-MP.

Salmon fillets (10 g aliquots) were spiked with either PA12 suspension or a mixture of six MP suspensions. Particle counts of PA12 and mixed MP suspensions indicated that each sample was spiked with 17–97 MP (RSD = 62%) or 292–895 MP (RSD = 49%) respectively. Zero to 92 MP (RSD = 170%) were counted in native salmon samples (10 g aliquots) whereas 1–164 MP (RSD = 135%) and 90–1281 MP (RSD = 83%) were counted in PA12-spiked and MP mixture–spiked samples respectively. Due to the high variations, no significant differences between spiking suspensions and spiked samples were observed regarding MP counts (unpaired Student’s *t*-test, *α* = 0.05), and particle size distribution (unpaired Wilcoxon-test, *α* = 0.05). Mass estimates of spiking suspensions and spiked samples were in the same order of magnitude with median values of 0.14 µg/sample, and 0.11 µg/sample for the PA12 series as well as 88 µg/sample, and 165 µg/sample for the MP mixture series, respectively. However, also high variations were observed in both spiking suspensions and spiked samples (RSD = 83–159%) regarding mass estimate.

### Performance of fluorescence microscopy in comparison with infrared spectroscopy

#### MP counting and characterisation

Only particles larger than 10 µm were considered due to the lower resolution of LDIR. Recovery rates were not determined, as samples could not be filtered quantitatively due to filter clogging. As a major proportion of the digested sample solution was discarded, especially low-density polymers (PE, PP, PS) floating at the surface were lost.

A total of 10,820 (sample 1) and 14,692 (sample 2) particles were counted with LDIR, and 72 and 161 particles were identified as MP (Table S10), respectively. All relevant polymer types were detected except from PA6, which could not be differentiated from natural peptides originating from matrix residues. Furthermore, residual fatty acids from the matrix could interfere with the characterisation of “rubber”; thus, those particles were also excluded. Fluorescence analysis detected fewer total particle numbers (92 and 610) but similar MP counts (23 and 124 MP).

#### Sample throughput

Imaging a Ø 47 mm filter surface (resolution 5 µm) required 15 ± 3 min per sample on average (1 *SD, n* = 60). Imaging with 1 µm resolution required approximately 30 min for a Ø 25 mm filter surface and was not feasible for a larger surface area due to the large data size (≥ 300 GB per image). An additional 13 ± 12 min was required for semi-automated image analysis (median 5 min; maximum 48 min). The high variability of time required for binary image generation was attributed to large amounts of matrix residues, particle agglomeration in highly loaded samples, high background fluorescence, or unevenly stained objects (e.g. fibres, crystalline MP like PET) requiring thorough manual particle selection to avoid over- or underestimation.

Due to the high particle numbers (*n* > 10,000), LDIR analysis required approximately 24–72 h per sample on an observed area of 2.3 cm^2^. Fluorescence analysis of the same samples required roughly 15 min per sample and was independent of total particle numbers (observed area 4.9 cm^2^). Fluorescence imaging was therefore significantly faster compared to LDIR imaging when analysing samples with high total particle numbers with a total speed of imaging and analysis of 0.02–0.06 h/cm^2^ (5 µm resolution) compared to 4.5–14.7 h/cm^2^ (10 µm resolution).

## Discussion

### Challenges of quantitative MP spiking

The complex nature of MP (broad range of size and morphology, various chemical compositions of polymer types and additives, different ageing states) is challenging for any analytical method [11]. Further challenges are posed by preparing complex matrices like seafood while preventing procedural contamination [13, 35, 40]. These factors contributed to the difficulties faced within the present study when determining MP recovery in spiked fish fillets especially regarding high deviations of particle counts in between samples as well as spiking suspensions.

One factor contributing to high deviations was the MP occurrence in native seafood samples, as it is difficult to obtain a guaranteed MP-free fish fillet matrix. In the present study, MP counts of 10 g aliquots of native salmon and herring fillet were highly variable (RSD > 100%), ranging from 0 to 92 MP/sample. The same amount of fillet was spiked with low particle numbers (17–97 MP, PA12) to analyse recovery at realistic concentration levels. However, random MP distribution in the native sample might have even exceeded the number of spiked MP, indicated by the high RSD (135%). Higher particle numbers were spiked with the MP mixture (292–895 MP), achieving a lower RSD (83%). The high variation of MP counts in native samples further indicates the difficulty of proper sample homogenisation for analysing non-soluble, microscale analytes. Larger sample aliquots might be needed in future studies for achieving more reproducible results. However, significantly increased sample sizes also lead to further challenges for quantitative sample preparation [35]. For example, increasing the temperature to achieve sufficient sample digestion results in negative impacts on polymer integrity (e.g. PET).

Another factor attributing to the deviation in spiked samples was the application of several MP suspensions per sample. As the densities of the polymers ranged from *ρ* ≈ 0.9 g/cm^3^ for PE(LD) to *ρ* ≈ 1.4 g/cm^3^ for PVC, each polymer type was suspended in a solution of different densities to prevent quick sedimentation or flotation. Particle numbers in aliquots of MP suspensions already differed within an RSD range of 12–34%. Due to polymer identification being infeasible, MP could only be determined as a sum parameter, resulting in an accumulation of individual errors. Current studies on developing MP reference material for interlaboratory comparison tests indicated that high heterogeneity between samples can occur. The RSD of particle numbers of MP-loaded capsules or reconstituted salt tablets (dissolved in water) ranged between 12 and 24% [41, 42]. Martínez-Francés et al. [42] observed higher deviations for particles below 50 µm, which was also indicated in this study for example when comparing PA6 (Ø 10–50 µm, RSD = 30%) with PVC (Ø 20–350 µm, RSD = 17%). This highlighted the difficulty of reproducible sample spiking for seafood method validation and standardisation. However, the homogeneity of MP in the solid salt tablets was lower compared to the reconstituted salt tablets (RSD = 8%), as the solid carrier matrix immobilised MP during handling and transport [41]. The same was observed comparing spiking reproducibility with soda tablets instead of soluble capsules (RSD = 9% for mixed MP in soda tablets) [42]. Consequently, spiking food matrices with solid MP carriers like salt tablets as opposed to particle dispersions should be considered in the future. Another promising approach for number-based sample spiking was described by Hildebrandt et al. [38] using laser microdissection pressure catapulting. The method demonstrated the capability to transfer exact particle numbers down to singular particles of PE, PET, and PS ranging between 10 and 16 µm in size. Further research is required for providing homogeneous reference material of seafood, spiked with MP of environmentally relevant size, morphology, and chemical composition.

### Detection and differentiation of particles from synthetic and natural origin for seafood analysis

So far, no sample preparation methods were described that are capable of destroying organic matrix completely without damaging plastics as well [27, 35]. Current methods for digesting organic-rich samples such as seafood therefore compromise between thorough matrix destruction and sustaining polymer integrity [43]. Thus, applying NR staining for these matrices poses a risk in terms of overestimating MP occurrence due to interfering PNO [27, 29]. Consequently, NR staining was rarely used for biota samples, so far [44]. MP counting in mussels revealed up to 14 times higher particle numbers when analysing with the NR-staining method as opposed to conventional FTIR analysis [28]. Within the present study, this challenge was overcome by optimising MP staining and implementing automated MP identification with RGB-based fluorescence threshold values.

Bright MP fluorescence is required for differentiation from PNO and to avoid under- or overestimation [29, 45]. The brightest fluorescence was achieved when MP was stained with dye-solvent solutions that induce polymer swelling. This can be attributed to the diffusion of the solvent into the polymeric network which facilitates the polymer-dye interaction [46, 47]. To account for the broad chemical and structural variety of MP potentially occurring in food and improve differentiation of MP and PNO, samples were therefore stained with two different NR solutions consecutively. As indicated in Fig. 2, hydrophobic polymers like PE or PP were stained more intensely by non-polar dye-solvent solutions (e.g. NR-hexane). Hydrophilic polymers like PA6, PET, or aged MP were stained more intensely by polar dye-solvent solutions (e.g. NR-EtAc). The swelling (and dissolution) behaviour of MP is very complex, for example depending on chemical composition, molecular mass, or the degree of crosslinking [46]. Of all polymers tested in the present study, PS was most sensitive to dissolution. Staining of BAM-PS with the optimised procedure revealed a reduction of particles smaller than 50 µm by 55% and simultaneously an increase of larger particles by 140% compared to staining with NR dissolved in isopropanol (illustrated in Fig. S15). This was attributed to the partial dissolution of several small particles in close range “melting” into large singular particles. The upper left picture of Fig. 2 shows barely distinguishable outlines of former particles merged. As this could not be differentiated by automated image analysis, the particle size distribution consequently was skewed towards larger particles. In contrast, staining with isopropanol did not affect the particle morphology noticeably, but resulted in diminished fluorescence (70% lower TPB compared to the optimised protocol) as described in “Impact of sample preparation on polymer integrity and fluorescence”. Diminished MP fluorescence can result in an underestimation of particles when their fluorescence cannot be differentiated from fluorescent PNO unambiguously (e.g. fishbone, chitin). While staining with isopropanol was sufficient for identifying PS, it was insufficient for detecting PE (Table S4). Alongside PP, PE is predominant among MP occurring in food, being reported in 96% of studies (*n* = 23). In contrast, PS was reported only in 35% of studies [48]. Consequently, proper PE detection is suggested to have a greater impact on particle counts of non-targeted MP analysis in seafood compared to a skewed size distribution attributed to partial PS dissolution. Nevertheless, when morphological data of PS are of specific concern, staining with isopropanol (or other alcohols) is recommended despite the underestimation of other seafood-relevant polymers.

The sensitivity of MP detection and identification was further limited by the influence of particle morphology or additives on fluorescence which is specific for the respective material (e.g. non-translucent black or blue particles). However, also conventional methods for MP identification, like Raman or FTIR, face these challenges. Black or fluorescent particles are also known to interfere with Raman- or infrared spectroscopy [49]. Ageing, additives, fillers, or colouring agents can alter or mask the polymer spectrum, hindering identification with spectra libraries [11, 50]. Ageing of MP can also impact their quantification, e.g. with thermoanalytical methods, by decreasing signals of pyrolysis products [51]. Due to the broad variety of MP occurring in the environment and in food samples, underrepresentation of certain types of MP may therefore occur with any non-targeted approach for MP analysis.

The protocol proposed in the present study achieved the classification of MP suspect particles (non-aged, pigment-free) and differentiation from seafood-related interferences with an average accuracy of 98 ± 2% (ß-error 2 ± 2%, α-error ≤ 1%). This is in accordance with the results provided by Meyers et al. [18], who achieved an accuracy of 94% for plastic classification with MP extracted from spiked mussels. Particle numbers observed in spiked fish fillet samples did not differ significantly from particle counts of the spiking solution, demonstrating the efficiency of implementing threshold values. Their efficiency was further confirmed by comparison with LDIR analysis. LDIR detected many particles (*n* = 10,000 per sample) but less than 2% were MP (72 MP, 161 MP). MP counts of fluorescence imaging were in the same order of magnitude (23 MP, 134 MP), confirming that MP occurrence was not significantly overestimated with semi-automated fluorescence imaging, contrary to other approaches with NR staining [28].To further improve differentiation of MP and PNO in environmental samples, counterstaining, i.e. the application of additional dyes like Calcofluor white, Evans blue, and 4ʹ,6-Diamidin-2-phenylindol (DAPI), was recommended. However, for optimal data processing, both blue (405 nm) and cyan (488 nm) excitations were required [29, 36], resulting in an increased analytical complexity. Counterstaining was not strictly needed for differentiating MP and PNO in the present study due to the implemented fluorescence threshold values, but featured more straightforward binary image generation due to a reduction of background fluorescence. As residual Calcofluor white or Evans blue did not interfere with MP detection, removing excess dye from the filter was not required. Therefore, counterstaining can be applied with minimal effort when initial fluorescence analysis reveals high background fluorescence or if the occurrence of interfering PNO (e.g. chitin or coloured cotton fibres) is likely.

### Potential of semi-automated fluorescence imaging as MP screening method

The most prominent advantage of NR staining coupled with (semi-)automated image analysis was the low analytical complexity and short analysis time of 20–60 min for MP screening in seafood depending on background fluorescence (17.4 cm^2^, 5 µm resolution, < 0.1 h/cm^2^). The application of machine learning tools and sophisticated automated particle detection may offer further potential to improve sample throughput with fluorescence imaging as the manual separation of particles and background for generating binary images was the most laborious step. Meyers et al. [18] trained a model for particle identification with RGB data of stained MP, PNO, and background fluorescence. They separated particles from the background by setting colour threshold values which required roughly 20 min per sample filter (47 mm diameter) [18]. This step required up to 48 min in the present study when samples had a high background fluorescence, e.g. due to large amounts of residual fishbone, tissue, or fat (fish oil). An interlaboratory comparison study on MP in drinking water indicated analysis times of 16 ± 26 h for visual microscopy, 10 ± 9 h for FTIR, and 19 ± 2 1 h for Raman analysis per sample, depending on how many particles were detected and identified respectively [52]. Even specialised techniques for fast mapping, like stimulated Raman scattering, require several hours (5 h/cm^2^) [53]. Due to the high numbers of particle-like matrix residues on the filter surfaces (*n* > 10,000), analysis of seafood samples with LDIR required 24–72 h per sample in the present study (2.3 cm^2^ filter area, 10 µm resolution; 4.5–14.7 h/cm^2^) which is comparable to conventional approaches.

MP occurrence in edible seafood tissue is low (0.02–6 MP/g) [6] and MP counts of the present study indicated higher reproducibility when using larger sample aliquots (RSD decreased by 27% when analysing 10 g instead of 1 g spiked herring fillet). As fluorescence imaging is compatible with a broad range of filter sizes and materials (e.g. cellulose [22], aluminium oxide [35], PTFE, glass fibre), sample aliquots of ≥ 10 g seafood can be analysed with minimal effort. In contrast, many spectroscopic methods require the application of tailor-made filter materials (e.g. gold-coated membranes) [13], which are often not suited for filtering large quantities of sample digestate. Consequently, particles would have to be pre-concentrated and transferred, which is not only laborious, but also prone to procedural contamination [35].

Semi-automated fluorescence imaging after NR staining therefore might be a valuable screening tool for improving sample throughput with conventional methods, as already recommended for µ-Raman analysis of environmental samples [54]. The proposed method can further supplement mass-based methods for MP analysis. Recovery rates of 62–111% were achieved with mass estimation of pure MP in suspensions (Table 1), indicating that mass estimation based on particle size and morphology achieves results in the same order of magnitude. When using only mass-based methods, a broad range of particle sizes present in a sample may interfere with quantification. Low numbers of large MP can mask the presence of small particles or even result in detector saturation due to the significantly higher particle mass, resulting in sample loss [11]. Considering the complexity of preparing organic-rich matrices like seafood for MP analysis, sample loss is a significant challenge. NR staining does not interfere with polymer identification when using mass-based methods like Py-GC/MS or NMR (data not shown), allowing for a simple implementation of the proposed method in consecutive analysis with mass-based methods. MP screening may be applied for determining potential interferences of large MP or if further dilution of the sample is necessary before applying destructive methods like Py-GC/MS.

## Conclusions

Large-scale MP analysis in the context of environmental and food screening is often limited by resource restraints due to the low sample throughput of conventional analytical methods. Fast screening methods are required to complement conventional approaches for particle-based analysis like FTIR and Raman spectroscopy or mass-based analysis like Py-GC/MS. The proposed seafood method achieved complete sample analysis (MP counting and mass estimation) within 1 h, demonstrating a higher sample throughout compared to conventional optical approaches. Due to the simple sample preparation and low impact of staining on polymer integrity, the proposed method can assist more sophisticated approaches by screening for MP occurrence in seafood.

Microscopic techniques often rely on high operator experience for reliable particle detection and characterisation, especially in organic-rich samples like seafood, due to the co-occurrence of matrix-inherent particles. The presented method minimised operator bias and MP overestimation by establishing threshold values for particle fluorescence. Like with other methods for MP analysis, not all plastics (e.g. pigmented or aged) can be identified equally. The proposed protocol unequivocally differentiated non-aged and non-pigmented MP of widely used polymers from seafood-related PNO. Of all tested MP, only the integrity of PS was affected to a recognisable extent. Due to the high variations of MP counts between samples, attributed to MP occurrence in native fish fillets and inhomogeneous sample spiking, the method’s suitability for MP quantification could not yet be fully evaluated. Further research on the method’s repeatability and robustness with more homogenously distributed and certified seafood reference material is needed.

### Supplementary Information

Below is the link to the electronic supplementary material.

Supplementary file1 (DOCX 2448 KB)
