# Supplementary material for: Nile red staining for rapid screening of plastic-suspect particles in edible seafood tissues
Source: Anal Bioanal Chem. 2024 May 10;416(14):3459–71. doi: 10.1007/s00216-024-05296-8 (PMC11106118; doi:10.1007/s00216-024-05296-8)
Supplement: Supplementary file 1 — Supplementary file1 (DOCX 2448 KB) [file 216_2024_5296_MOESM1_ESM.docx]

Supplementary Information

**Nile red-staining for rapid screening of plastic-suspect particles in edible seafood tissues**

Julia Süssmann^1^, Elke Kerstin Fischer^2^, Lars Hildebrandt^3^, Elke Walz^4^, Ralf Greiner^4^, Sascha Rohn^5^, Jan Fritsche^1*^

^1^Max Rubner-Institut, Federal Research Institute of Nutrition and Food, Department of Safety and Quality of Milk and Fish Products, Hermann-Weigmann-Straße 1, 24103 Kiel, Germany

^2^University of Hamburg, Center for Earth System Research and Sustainability (CEN), Bundesstraße 55, 20146 Hamburg, Germany

^3^Helmholtz-Zentrum hereon, Institute of Coastal Environmental Chemistry, Department of Inorganic Environmental Chemistry, Max-Planck-Straße 1, 21502 Geesthacht, Germany

^4^Max Rubner-Institut, Federal Research Institute of Nutrition and Food, Department of Food Technology and Bioprocess Engineering, Haid-und-Neu-Straße 9, 76131 Karlsruhe, Germany

^5^Technische Universität Berlin, Institute of Food Technology and Food Chemistry, Department of Food Chemistry and Analysis, TIB 4/3-1, Gustav-Meyer-Allee 25, 13355 Berlin, Germany

*****Corresponding author
E-mail address: [jan.fritsche@mri.bund.de](mailto:jan.fritsche@mri.bund.de)

Table of contents

[1 Supplemental information on materials 3](#_Toc163567420)

[1.1 Preparation of reference material from particles of synthetic and natural origin 3](#_Toc163567421)

[2 Supplemental information on the characterisation of fluorescent particles 5](#_Toc163567422)

[2.1 Additional instrumental analyses 5](#_Toc163567423)

[2.1.1 Analysis with confocal laser scanning microscopy 5](#_Toc163567424)

[2.1.2 Analysis with fluorescence microscopy (FITC-filter cube) 5](#_Toc163567425)

[2.2 Image processing and analysis 5](#_Toc163567426)

[2.2.1 Calculation of particle shape 5](#_Toc163567427)

[2.2.2 Calculation of particle size 7](#_Toc163567428)

[2.2.3 Calculation of particle mass 8](#_Toc163567429)

[2.2.4 Determination of polymer type by fluorescence 8](#_Toc163567430)

[2.3 Impact of chemical treatment on polymer characterisation 12](#_Toc163567431)

[2.3.1 Impact of sample digestion 12](#_Toc163567432)

[2.3.2 Impact of counterstaining 12](#_Toc163567433)

[2.4 Impact of particle-inherent properties on polymer characterisation 13](#_Toc163567434)

[2.4.1 Impact of particle morphology 13](#_Toc163567435)

[2.4.2 Impact of particle colour on fluorescence 14](#_Toc163567436)

[2.4.3 Impact of artificial UV-aging on polymer classification 15](#_Toc163567437)

[2.5 Optimisation of staining and measurement conditions for the application to seafood 16](#_Toc163567438)

[2.5.1 Assessment of dye concentration, incubation time, and temperature 16](#_Toc163567439)

[2.5.2 Influence of solvent choice on fluorescence 17](#_Toc163567440)

[2.5.3 Impact of staining on polystyrene particles 20](#_Toc163567441)

[2.5.4 Evaluation of excitation wavelengths 21](#_Toc163567442)

[3 Supplementary information on particle recovery 24](#_Toc163567443)

[3.1 Procedural contamination 24](#_Toc163567444)

[3.2 Comparison of fluorescence imaging and LDIR imaging particle counting 26](#_Toc163567445)

[4 References 27](#_Toc163567446)

# Supplemental information on materials

## Preparation of reference material from particles of synthetic and natural origin

Commercially relevant plastic particles and nurdles [1] were purchased from Goodfellow Cambridge Ltd. (Huntingdon, United Kingdom) and Alfa Aesar GmbH & Co KG (Karlsruhe, Germany), namely nylon 6 (PA6), nylon 12 (PA12), polyacrylonitrile (PAN), polycarbonate (PC), low-density polyethylene (PE(LD)), polyethylene terephthalate (PET), polypropylene (PP), polystyrene (PS), polysulfone (PSU), polytetrafluoroethylene (PTFE), polyurethane (PU), and polyvinylchloride (PVC) as listed in Table S1, Nos. 1–13. When necessary, polymers were milled with an ultracentrifugal mill (ZM100, Retsch GmbH, Haan, Germany) after cooling with liquid nitrogen [2] and consecutive sieving with stainless-steel meshes to obtain particles of roughly 100 µm – 200 µm size. Additionally, small PP particles were obtained after dissolution in boiling toluene, precipitation after cooling, and consecutive sieving [3].

In order to evaluate the selectivity of NR-staining, the six polymers occurring most commonly as MP in the environment [4] were chosen for further testing, e.g. regarding the impact of pigments, surface morphology, or UV-degradation, namely PA6, PE, PET, PS, and PVC.

UV-aged polymers were obtained by spreading a thin layer of MP particles in Petri dishes and irradiating them in a UV-Box for 26 h and 72 h respectively (λ ≈ 365 nm, 8x 12 Watt). Taking the solar constant of 1362 W/m² and the size of the Petri dishes (9 cm diameter) into account, this would approximate to a sun exposure of 12 and 35 days at sea near the equator.

Another set of plastic particles with a size predominantly smaller than 100 µm were provided by the Bundesanstalt für Materialforschung und -prüfung (BAM, Berlin, Germany; Table S1, Nos. 14–19).

Small particles of coloured plastics were obtained from household items (Table S1, Nos. 20–26) cut with a scalpel, ground in a mortar, or milled with a coffee mill. Small fibres were obtained by cutting yarn (cotton, polyester, nylon) and isolating singular fibres with tweezers.

PNO plausible to occur in seafood samples (Table S1, Nos. 27–31) were prepared from fishbone, bivalve shells, and shrimp shells (carapace and abdominal segments) after removing adhering tissue, drying and grinding. Furthermore, purified sea sand was purchased by Th. Geyer GmbH & Co. KG (Renningen, Germany).

To avoid potential polymer dissolution by certain solvents [5], a solution of 1 mg/mL NR in isopropanol [6] was prepared for staining. Particles or fibres were suspended in NR solution and the solvent was left to evaporate at room temperature. Dried particles or fibres were placed onto glass fibre filters (GFF, 1.2 µm particle retention; Th. Geyer GmbH & Co. KG, Renningen, Germany). For transport and analysis, filters were placed between glass slides and fixed with adhesive tape. The samples were then analysed with fluorescence and confocal laser scanning microscopy to establish threshold values for differentiation.

**Table S1** Materials used for establishing threshold values for polymer differentiation depending on particle fluorescence after NR-staining. Polymers used for optimisation of the staining protocol and determining threshold values for polymer identification are underlined (referred to as “in-house reference material”)

| **No.** | **polymer** | **details** | **provider / source** | **provided size** | **application form** |
| --- | --- | --- | --- | --- | --- |
| 1 | PA6 | particles | Goodfellow | 10 – 50 µm | as provided, UV-aged, spiking-suspension |
| 2 | PE(LD) | particles | AlfaAesar | 500 µm | as provided, UV-aged, spiking-suspension |
| 3 | PET | particles | Goodfellow | < 300 µm | as provided, UV-aged, spiking-suspension |
| 4 | PP | nurdles | Goodfellow | 4 mm | cryomilled (100 – 200 µm), UV-aged (cryomilled), spiking-suspension (cryomilled), precipitated (100 – 200 µm) |
| 5 | PS | rod, cross-linked | Goodfellow | 6 x 2 mm | cryomilled (100 – 200 µm), UV-aged (cryomilled), spiking-suspension (cryomilled) |
| 6 | PVC | particles | Goodfellow | < 250 µm | as provided, UV-aged, spiking-suspension |
| 7 | PA6 | nurdles | Goodfellow | 3 mm | cryomilled (100 – 200 µm) |
| 8 | PA12 | particles | Goodfellow | 10 – 50 µm | as provided |
| 9 | PAN | particles | Goodfellow | 50 µm | as provided |
| 10 | PC | nurdles | Goodfellow | 3 mm | cryomilled (100 – 200 µm) |
| 11 | PSU | nurdles | Goodfellow | 3 mm | cryomilled (100 – 200 µm) |
| 12 | PU | nurdles | Goodfellow | 3 – 5 mm | cryomilled (100 – 200 µm) |
| 13 | PTFE | particles | Goodfellow | < 675 µm | as provided |
| 14 | PA6 | particles, weathered | BAM | 20 – 500 µm | as provided |
| 15 | PE(LD) | particles | BAM | 5 – 70 µm | as provided |
| 16 | PET | particles | BAM | 10 – 200 µm | as provided |
| 17 | PP | particles | BAM | 100 – 450 µm | as provided |
| 18 | PS | particles | BAM | 90 – 300 µm | as provided |
| 19 | PVC | particles | BAM | 50 – 100 µm | as provided |
| 20 | PP | particles (red, black) | bottle cap | - | ground (100 – 500 µm),  precipitated (100 – 200 µm) |
| 21 | PP | film, coloured | book protection film | - | cut (500 – 1000 µm)  (red, blue, green, yellow, transparent) |
| 22 | PS | particles (black) | food container | - | ground (100 – 500 µm) |
| 23 | PS | foam / expanded | packaging | - | cut (500 – 1000 µm) |
| 24 | PA6 | fibres, transparent | fishing line | 3 mm width | cut & tweezered (500 – 1000 µm length) |
| 25 | PET | fibres, coloured | polyester yarn | - | cut & tweezered (500 – 1000 µm length) (white, red, orange, yellow, green, blue, purple, black) |
| 26 | cellulose | fibres, coloured | cotton yarn | - | cut & tweezered (500 – 1000 µm length)  (white, red, orange, yellow, green, blue, purple, black) |
| 27 | cellulose | paper tissue | laboratory | - | cut & tweezered (500 – 1000 µm) |
| 28 | chitin | shrimp shells | matrix | - | ground (100 – 200 µm) |
| 29 | fishbone | salmon carcass | matrix | - | ground (100 – 200 µm) |
| 30 | sea sand | acid-purified, calcined | Th. Geyer | 100 – 300 µm | as provided |
| 31 | seashell | blue mussel shells | matrix | - | ground (100 – 200 µm) |

# Supplemental information on the characterisation of fluorescent particles

## Additional instrumental analyses

### Analysis with confocal laser scanning microscopy

For confocal laser scanning microscopy, samples were analysed with a Leica TCS SP8 inverse confocal microscope (Leica Microsystems GmbH, Wetzlar, Germany) equipped with lasers of the wavelengths 405 nm, 488 nm, 552 nm, and 638 nm using a 10x-objective. Emission spectra were acquired in the wavelength range of 500–790 nm (excitation wavelength *λ_ex_* = 405 nm, *λ_ex_*= 488 nm), in the range of 560–590 nm (*λ_ex_* = 552 nm) and in the range of 650–790 nm (*λ_ex_* = 638 nm). To evaluate the feasibility of polymer differentiation based on fluorescence brightness and colour, a filter area of approximately 35 mm² was scanned (resolution 1 µm) using the 488 nm laser (10 % intensity, gain 500, offset 0 %) and measuring emission in the range of 525–545 nm (green light), 595–605 nm (yellow light), and 610–620 nm (orange light). A stack of 100 µm distance (10 µm-steps) was measured and reduced to 2D-data using maximum projection, for obtaining a sharp image of the particles due to the low depth of field.

### Analysis with fluorescence microscopy (FITC-filter cube)

A subset of samples was also analysed with the Leica TCS SP8 for observing particle fluorescence with a FITC-filter set (460–490 nm excitation wavelength, >510 nm emission wavelength). As the microscope was not equipped with a colour camera, colour images were taken via the ocular with a smartphone camera (iPhone SE2020, automated exposure deactivated).

## Image processing and analysis

### Calculation of particle shape

For shape determination, common shape categories for MP characterisation, namely spheroids, fragments, and fibres, were used [7]. They were further differentiated into spherical, rounded, angular, and irregular particles as well as elongated and coiled fibres based on particles shapes observed in preliminary MP analysis of seafood products. 100 particles of each shape associated with the respective category were hand-drawn in different sizes (Fig. S1) using Adobe Photoshop CC. Shape descriptors were calculated according to the equations listed in Table S2. This included shape descriptors implemented in ImageJ (circularity, aspect ratio, solidity, roundness), shape descriptors recommended for image analysis in general (elongation, convexity) [8-12], descriptors established for MP identification in soil [13] (compactness) as well as the ratio of bounding rectangle and convex hull. Relevant threshold parameters for shape classification were determined empirically by maximising the number of true positive classification and minimising false positive classification for each shape. A classification order to achieve minimal false-positive results was established, namely rounded → spherical → irregular → angular → coiled fibres → elongated fibres. The classification system was then applied to the particle images for assessing type I-errors (false-positive) and type II-errors (false-negative) for each 2D-shape as well as 3D-type category.

| 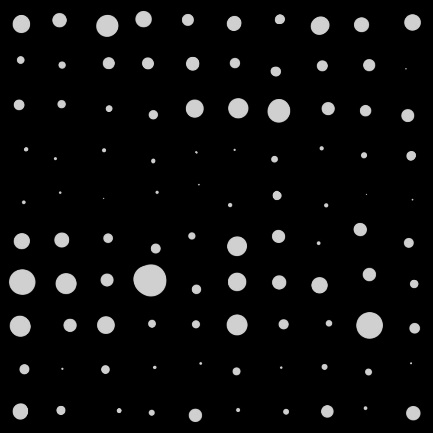 | 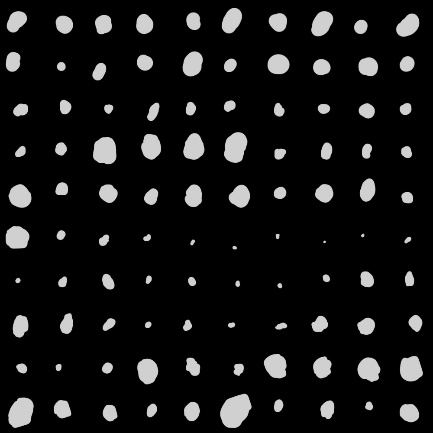 | 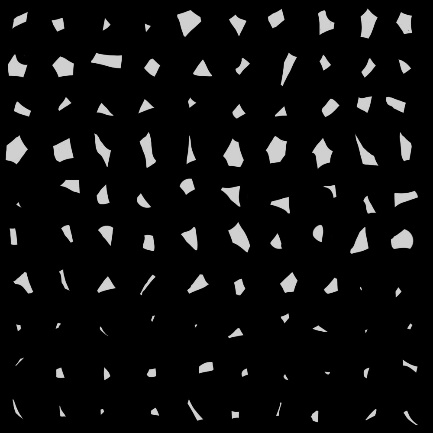 |
| --- | --- | --- |
| 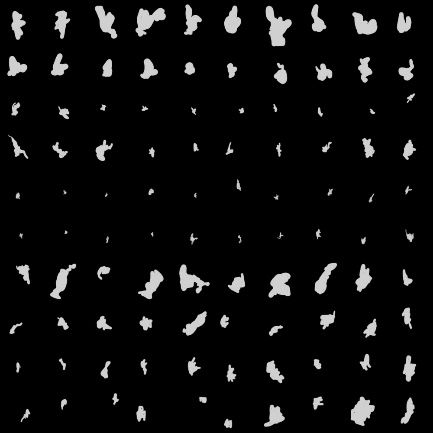 | 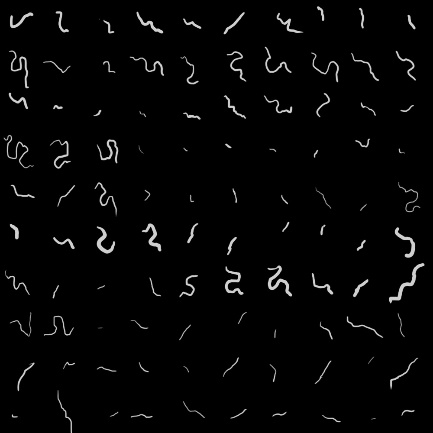 | 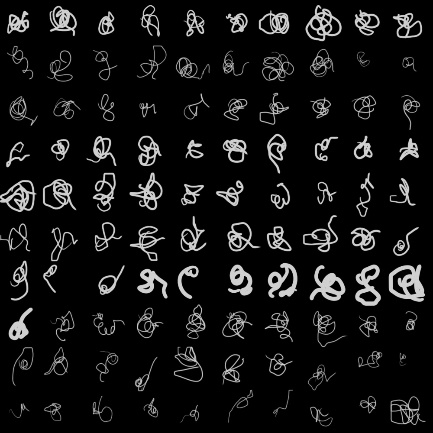 |

**Fig. S1** Illustrations of particles (n = 100) associated with the shape description of “spherical”, “rounded”, “angular”, “irregular” particles as well as “elongated” and “coiled” fibres (from top left to bottom right).

**Table S2** Description and equations of parameters evaluated for describing MP shape

| **parameter** | **description** | **equation** | **ref.** |
| --- | --- | --- | --- |
| circularity | similarity of a shape to a circle and ranges from 0.0 to 1.0 with a circularity of 1.0 for a perfect circle | $\text{circularity}\text{ }\text{=}\text{ }\text{4π}\text{ }\text{∙}\text{ }\frac{\text{particle area}}{\text{perimeter}}$ | [13] |
| aspect ratio | ratio of the major and minor axis of the best fitting ellipse surrounding the particle | $\text{aspect ratio =} \frac{\text{major axis}}{\text{minor axis}}$ | [13] |
| solidity | measure for the extent of convexity or concavity of a shape; calculated as the quotient of particle area and the area of its convex hull (CH) | $\text{solidity}\text{ }\text{=}\text{ }\frac{\text{particle area}}{\text{CH area}}$ | [8]  [12] |
| roundness | circularity of a particle corrected by its aspect ratio | $\text{roundness}\text{ }\text{=}\text{ }\frac{\text{4}\text{ }\text{∙}\text{ }\text{particle area}}{\text{π}\text{ }\text{∙}\text{ }\text{major axis}}$ | [11] |
| compactness | 2D geometric shape descriptor for MP in soils | $\text{compactness}\text{ }\text{=}\text{ }\frac{\left( \frac{\text{4}}{\text{π}} \right)^{\text{2}}}{\text{major axis}}$ | [13] |
| elongation | ratio between the length and width of the bounding rectangle (BR) | $\text{elongation}\text{ }\text{=}\text{ }\frac{\text{height}_{\text{BR}}}{\text{length}_{\text{BR}}}$ | [9] |
| convexity | ratio of the perimeters of the convex hull and the particle itself | $\text{convexity = }\frac{\text{perimete}\text{r}_{\text{CH}}}{\text{perimeter}}$ | [8] |
| perimeter of convex hull | approximated to the perimeter of the best fitting ellipse (Ramanujan approximation) with “a” and “b” describing half of the axis of the best fitting ellipse respectively | $\text{perimete}\text{r}_{\text{CH}}\text{ }\text{≈}\text{ }\text{(a}\text{ }\text{+}\text{ }\text{b)}\text{ }\text{∙}\text{ }\text{π}\text{ }\text{∙}\text{ }\left( \text{1+}\frac{\text{3}\text{ }\text{∙}\text{ }\left( \frac{\text{a}\text{ }\text{-}\text{ }\text{b}}{\text{a}\text{ }\text{+}\text{ }\text{b}} \right)^{\text{2}}}{\text{10}\text{ }\text{+}\text{ }\sqrt{\text{4}\text{ }\text{-}\text{ }\text{3}\text{ }\text{∙}\text{ }\left( \frac{\text{a}\text{ }\text{-}\text{ }\text{b}}{\text{a}\text{ }\text{+}\text{ }\text{b}} \right)^{\text{2}}}} \right)$ | [10] |
| area ratio | proportion of the particle’s convex hull area to the area of the bounding rectangle | $\text{area ratio}\text{ }\text{=}\text{ }\frac{\text{CH}\text{ }\text{area}}{\text{ BR area}}\text{=}\frac{\text{particle area}}{\text{solidity}\text{ }\text{∙}\text{ }\text{widt}\text{h}_{\text{BR}}\text{ }\text{∙}\text{ }\text{lengt}\text{h}_{\text{BR}}}$ |  |

Threshold values and error rates of shape determination are listed in Table S3. The model classified more than 95 % of particles and more than 84 % of fibres correctly, indicated by error rates of false-negative classification (ß-error) of 5 % and 16 % or lower respectively. The model was prone to confusing short fibres with angular or irregular particles, as indicated by error rates of false-positive classification (α-error) between 11 % and 18 %. The model was further able to differentiate between spherical, rounded, and irregular particles with a probability of ≥ 93 % (ß-error ≤ 7 %). However, angular particles were mostly misclassified as irregular. Elongated and coiled fibres were differentiated with a probability ≥ 62 % (ß-error ≤ 38 %). With the exception of *aspect ratio*, all other shape descriptors contributed to differentiating between particle shapes. The *aspect ratio* described the ratio of major and minor axis of the best fitting ellipse and was used by Ismayilova, Zeyer and Timpf [13] for describing MP particle shape in soil samples. As this descriptor did not improve differentiation of particle shapes observed in seafood, the parameter was not considered further.

**Table S3** Threshold values suited for the characterisation of particle shapes (“-“: irrelevant for differentiation);
α-error – false-positive classification, ß-error – false-negative classification

|  | **spheroids** | | **fragments** | | **fibres** | |
| --- | --- | --- | --- | --- | --- | --- |
| **parameter** | **spherical** | **rounded** | **angular** | **irregular** | **elongated** | **coiled** |
| circularity | 0,90 – 1,01 | 0,75 – 0,94 | 0,27 – 0,84 | 0,31 – 0,84 | - | - |
| solidity | - | 0,90 – 0,97 | 0,63 – 0,95 | 0,51 – 0,93 | - | 0,16 – 0,70 |
| roundness | - | 0,47 – 0,97 | - | - | - | - |
| compactness | - | - | - | 0,02 – 0,21 | - | 0,02 – 0,13 |
| elongation | - | 0,59 – 1,00 | - | 0,38 – 0,98 | - | - |
| convexity | - | 0,92 – 1,02 | - | 0,58 – 0,95 | - | 0,20 – 0,72 |
| area ratio | - | 0,68 – 0,93 | - | 0,50 – 0,88 | - | 0,56 – 1,00 |
|  |  |  |  |  |  |  |
|  | performance of 2D-shape classification | | | | | |
| α-error | 5 % | 10 % | 15 % | 79 % | 25 % | 22 % |
| ß-error | 7 % | 5 % | 66 % | 7 % | 38 % | 23 % |
|  |  |  |  |  |  |  |
|  | performance of 3D-type classification | | | | | |
| α-error | 0 % | 3 % | 11 % | 18 % | 5 % | 0 % |
| ß-error | 0 % | 0 % | 5 % | 3 % | 16 % | 13 % |

### Calculation of particle size

The particle size of spheroids and fragments was determined according to Feret’s diameter [14] as provided by ImageJ. Due to fibres might be curled up, fibre width and length were calculated based on the particle perimeter and area which are independent from the placement [9] according to equations (1) and (2):

| $\text{fibre length}\text{ }\text{=}\text{ }\frac{\text{perimeter}\text{ }\text{- }\sqrt{\text{perimete}\text{r}^{\text{2}} \text{-}\text{ }\text{16}\text{ }\text{∙}\text{ }\text{particle area}}}{\text{4}}$ | (1) |
| --- | --- |
| $\text{fibre width}\text{ }\text{=}\text{ }\frac{\text{particle area}}{\text{fibre length}}$ | (2) |

### Calculation of particle mass

A rough estimate of the particle mass was calculated as the product of approximate density and particle volume. The polymer density ($\bar{\text{ρ}}$) was calculated based on the average (approximate) density of all polymers for which the particle was eligible based on its fluorescence (classified according to the threshold values described previously). The volume was calculated differently for each shape category according to equations (3) – (5). Fibres were approximated to a cylinder with fibre length describing the cylinder length and fibre width describing the diameter of the cylinder. The volume of spherical and rounded particles was approximated to the volume of a sphere using the maximum Feret diameter as a proxy for the sphere diameter. Irregular and angular particles were approximated to cuboids and the volume was calculated based on the particle area and the height of the particle. As no confocal information of the particles was available, the height was assumed to be approximate to the minimum Feret diameter based on the hypothesis, that particles pressed between glass slides would lay flat on the filter surface, so the particle height would not exceed the longitudinal size.

| $\text{cylindrical volume}\text{ }\text{=}\text{ }\frac{\text{π}\text{ }\text{∙}\text{ }\text{particle are}\text{a}^{\text{2}}}{\text{4 ∙}\text{ }\text{fibre length}}$ | (3) |
| --- | --- |
| $\text{spherical volume}\text{ }\text{=}\frac{\text{1}}{\text{6}}\text{π}\text{ }\text{∙}\text{ }{\text{Feret}_{\text{max}}}^{\text{3}}$ | (4) |
| $\text{cuboid volume}\text{ }\text{=}\text{ }\text{particle area ∙}\text{ }\text{Feret}_{\text{min}}$ | (5) |

### Determination of polymer type by fluorescence

Polymer types were determined by threshold values for total particle brightness and colour [16, 17]. Therefore, at least 100 particles of stained reference particles (PA6, PA12, PAN, PC, LDPE, PET, PP, PS, PSU, PU, PVC) and interfering PNO (chitin, cotton, fishbone, seashell) were analysed. Total particle brightness (TPB) was calculated as the sum of the mean pixel brightness of the red and green channel for fluorescence microscopy images or the sum of the three emission ranges (green, yellow, orange) for laser scanning microscopy. To assess colour independently from particle brightness, the proportion of red pixel brightness was calculated for fluorescence images. For laser scanning images, the proportion of brightness from the green and yellow wavelength range were calculated respectively. To account for 99.7 % of the respective particles, classification ranges using three times the standard deviation were calculated. Particles could classify as more than one polymer due to overlapping fluorescence parameters but only particles that did not classify as PNO were classified as MP suspect. MP suspect particles were grouped by “low”, “medium” and “high” based on brightness and colour. Low-group particles had a lower proportion of red fluorescence compared to PNO (low chemical polarity) like PE, or PP. High-group particles were of high chemical polarity with the highest brightness among reference particles like PVC or PU. All other MP suspect particles were classified as medium-group.

All MP used as in-house reference (Table S1, Nos. 1–12) were stained by NR. Among all considered synthetic materials, the brightest fluorescence was achieved for PS, PU, and PVC, while the lowest were for PA6, PE, and PP. As expected, sea sand was not stained by NR due to its inorganic composition and thus, showed no fluorescence. Even though seashells and fishbones mainly consist of inorganic constituents, residual proteins and lipids that can be present of up to 0.5 % attributed to particle fluorescence [18]. Fibres from cotton lab coats and cellulose tissues (potential procedural contamination) as well as ground shrimp carapace (chitin) exhibited fluorescence as well. Except for chitin, the fluorescence of PNO was weaker compared to that of synthetic polymers. Consequently, only detection of PE was affected, due to the weak fluorescence of the latter. The colour of emitted fluorescence differed between polymers based on their chemical polarity. Fluorescence of polar particles (e.g. PSU, PVC, fishbone) appeared red, whereas particles of non-polar polymers (e.g. PE, PE, PS) appeared green or yellow. The colour differences of polymers as observed when using a FITC filter are visualised in Fig. S2.


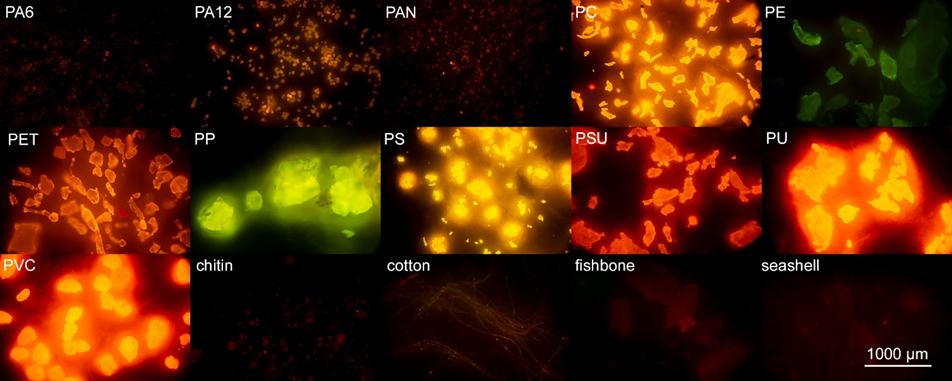


**Fig. S2** Photographs of selected in-house reference materials of polymers from synthetic and natural origin stained with 1 mg/mL Nile Red in isopropanol, observed with a FITC-filter set.

Different polymer groups were observed when plotting average values for TPB and proportion of red fluorescence of polymers observed with fluorescence microscopy as illustrated in Fig. S3 (upper graph). The application of 1.96-times the standard deviation (*SD*) indicated, that more than 95 % of all observed polymer particles could be differentiated from PNO except for PE, and PAN. However, particle fluorescence of singular particles varied greatly within polymer samples. Consequently, no polymer except from PU could be identified without fault. This was even more pronounced for laser scanning microscopy as indicated in Fig. S3 (lower graph). Here, though, differentiation of PA6 and PET was affected. In contrast, even though PE fluoresced weakly, it could be differentiated from PNO due to a high proportion of green fluorescence.

A mean ß-error (false negative classification) of 5±1 % (1 *SD*, *n* = 9) was achieved for MP suspect classification with fluorescence microscopy when excluding the extreme values of 28 % and 45 % for PAN and PE(LD). For classifying the polymer type, mean ß-errors of 24±17 % (1 *SD*, *n* = 12) were achieved. With laser scanning microscopy, a mean ß-error of 2±3 % was achieved (1 *SD*, *n* = 8), when excluding the extreme values of 61 %, 75 %, 21 % for PA6, PE(LD), and PP. Mean ß-errors of 61±28 % were achieved for polymer classification. A proportion of 1 % and 9 % of the chitin particles was misidentified as MP suspect (α-error) using fluorescence and laser scan microscopy, respectively. Details of the classification performance for each polymer are listed in Table S4.

Generally, the performance of polymer differentiation was higher, when using fluorescence microscopy even though laser scan microscopy offered more information regarding particle colour as also the green emission wavelength range was observed opposed to fluorescence microscopy. The lower performance of laser scan microscopy is assumed to be based on the higher variability of particle fluorescence. Fluorescence brightness observed with fluorescence microscopy was distributed more evenly across the particle surface, especially in the case of translucent polymers like PET, as shown exemplarily in Fig. S4.

**
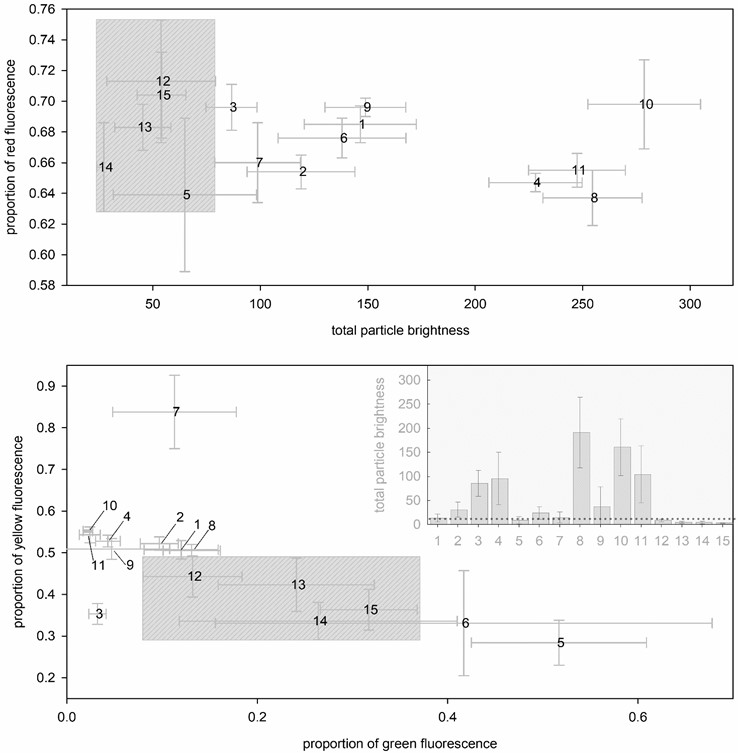
**

**Fig. S3** Average values (1.96 *SD, n* ≥ 100) of total particle brightness (TPB) and proportion of red, or green and orange fluorescence for polymer differentiation by fluorescence microscopy (upper graph) and laser scanning microscopy (lower graph). TPB for laser scanning microscopy is illustrated in the upper right corner of the lower graph. The hatched areas and the dotted line (lower graph, top right) mark interferences by PNO. 1 – PA6; 2 – PA12; 3 – PAN; 4 – PC; 5 – PE(LD); 6 – PET; 7 – PP; 8 – PS; 9 – PSU; 10 – PU; 11 – PVC; 12 – chitin; 13 – cotton; 14 – fishbone; 15 – seashell

**
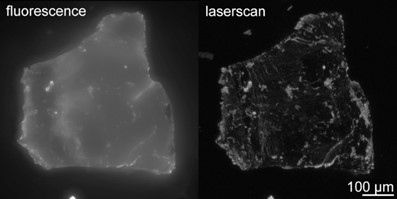
**

**Fig. S4** Photograph (FITC filter cube; left), and laser scanning image (λ_ex_ = 488 nm; right) of the same transparent PET-particle (NR-stained).

**Table S4** Error rates of plastic identification for in-house reference and BAM-MP after NR staining (with optimised staining method or NR dissolved in isopropanol). ß – proportion of false negative results (ß-error), α – proportion of false positive results (α-error)

| **method** | laser scanning | fluorescence microscopy | fluorescence microscopy | fluorescence microscopy |
| --- | --- | --- | --- | --- |
| **plastics** | in-house reference | in-house reference | in-house reference | BAM-MP |
| **staining** | isopropanol | isopropanol | optimised method | optimised method |
| PA6 | ß = 61 % | ß = 5 % | ß = 0 % | ß = 97 % |
| PA12 | ß = 0 % | ß = 5 % |  |  |
| PAN | ß = 0 % | ß = 28 % |  |  |
| PC | ß = 0 % | ß = 4 % |  |  |
| PE(LD) | ß = 75 % | ß = 45 % | ß = 1 % | ß = 75 % |
| PET | ß = 4 % | ß = 5 % | ß = 0 % | ß = 5 % |
| PP | ß = 21 % | ß = 3 % | ß = 4 % | ß = 61 % |
| PS | ß = 7 % | ß = 5 % | ß = 4 % | ß = 0 % |
| PSU | ß = 3 % | ß = 7 % |  |  |
| PTFE | no fluorescence | no |  |  |
| PU | ß = 0 % | ß = 5 % |  |  |
| PVC | ß = 0 % | ß = 7 % | ß = 0 % | ß = 100 % |
|  |  |  |  |  |
| chitin | α = 9 % | α = 1 % | α = 1 % |  |
| cotton | α = 0 % | α = 0 % | α = 0 % |  |
| fishbone | α = 0 % | α = 0 % | α = 0 % |  |
| seashell | α = 0 % | α = 0 % | α = 0 % |  |
| sea sand | no fluorescence | no |  |  |

**Table S5** Error rates of polymer type identification for in-house reference and BAM-MP after NR staining (with optimised staining method or NR dissolved in isopropanol). ß – proportion of false negative results (ß-error), α – proportion of false positive results (α-error)

| **method** | laser scanning | fluorescence microscopy | fluorescence microscopy | fluorescence microscopy |
| --- | --- | --- | --- | --- |
| **plastics** | in-house reference | in-house reference | in-house reference | BAM-MP |
| **staining** | isopropanol | isopropanol | optimised method | optimised method |
| PA6 | ß = 84 % | ß = 48 % | ß = 76 % | ß = 97 % |
| PA12 | ß = 67 % | ß = 37 % |  |  |
| PAN | ß = 79 % | ß = 13 % |  |  |
| PC | ß = 64 % | ß = 18 % |  |  |
| PE(LD) | ß = 53 % | ß = 4 % | ß = 85 % | ß = 75 % |
| PET | ß = 20 % | ß = 35 % | ß = 71 % | ß = 5 % |
| PP | ß = 84 % | ß = 50 % | ß = 68 % | ß = 61 % |
| PS | ß = 0 % | ß = 8 % | ß = 68 % | ß = 0 % |
| PSU | ß = 81 % | ß = 33 % |  |  |
| PU | ß = 66 % | ß = 4 % |  |  |
| PVC | ß = 77 % | ß = 14 % | ß = 9 % | ß = 100 % |

## Impact of chemical treatment on polymer characterisation

### Impact of sample digestion

Pure MP was treated with reagents used for seafood digestion (similar concentration, incubation temperature and time). No significant differences were observed in particle fluorescence before and after chemical treatment of MP (enzymatic-alkaline digestion, oxidative filter treatment).





**Fig. S5** Average total particle brightness (1 *SD*, *n*≥ 100) of MP before (dark grey) and after (light grey) sample clean-up (enzymatic-alkaline digestion and oxidative treatment). Hatched bars illustrate the respective average proportion of red fluorescence.

### Impact of counterstaining

Subsequent staining of NR-stained MP with Evans Blue dye or Calcofluor White staining agent resulted in overall decreased fluorescence but not to a significant extent as illustrated in Fig. S7. The colour of fluorescence was not affected. Due to the high variability of particle fluorescence, counterstaining did not improve the differentiation of natural and synthetic materials when applying the classification system.





**Fig. S6** Comparison on the average total particle brightness (1 *SD, n* ≥ 7) of NR-stained particles (hatched grey) compared to additional staining with Calcofluor White (dark grey) or Evans Blue dye (light grey).

## Impact of particle-inherent properties on polymer characterisation

### Impact of particle morphology

To assess whether an impact of particle morphology on particle fluorescence was indicated, data sets of particle brightness, size, and shape were checked for linear correlation. Therefore, the Pearson correlation coefficient (PCC) was calculated. Medium to strong correlation of TPB and particle size (Feret’s diameter) was observed for 53 % of samples (*n*= 43) with a PCC ≥ 0.4. Weak or no correlation was observed for all samples between TPB and the shape descriptors *circularity* and *roundness* (PCC ≤ 0.4). Furthermore, no correlation of particle colour (proportion of red brightness) with size or shape descriptors was observed.

When analysing transparent/white polymers of different origins, size and morphology affected particle fluorescence significantly. Statistical significance was calculated with Student’s *t*-test with a significance level of 5 % (α = 0.05). For example, particles in the size range from 50–100 µm from cryomilled PA6 nurdles (Table S1, No. 7) were significantly brighter and redder compared to similar sized particles of the in-house PA6-reference (Table S1, No. 1) with a mean TPB of 130±7 (1 *SD*, *n*= 25) and 75±11 (1 *SD*, *n*= 990) respectively (respective red proportion of 68.3±0.7 % and 66.9±0.7 %). In contrast, white/transparent nylon (PA6) and polyester fibres (Table S1, Nos. 24, 25) were stained to a lesser extent compared to the in-house reference of PA6 and PET. Consequently, no nylon fibres investigated in this study were classified as MP suspect, whereas only 5 % of PET fibres and 33 % of small PA6 particles were classified as MP suspect. This confirmed the findings of previous studies, especially regarding the fluorescence of fibres [32, 51].

Another factor contributing to particle brightness was surface roughness as exemplarily shown in Fig. S8. Precipitated PP particles with a rough surface fluoresced significantly brighter than ground particles with a comparatively smooth surface from the same source material (bottle cap).


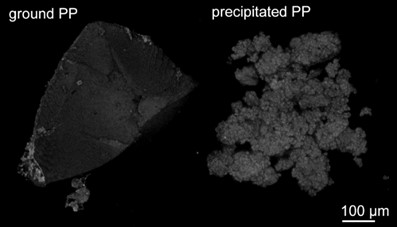


**Fig. S7** Laser scanning images (λ_ex_ = 488 nm) of NR-stained particles prepared from the same PP-bottlecap by grinding of large fragments (left) and precipitation from polymer solution (right)

### Impact of particle colour on fluorescence

Inherent particle colour had a major influence on particle fluorescence. Especially particles with dark colours like blue or black were in many cases not even marginally stained by NR (e.g. tire rubber, black PP caps, black polyester yarn) being in accordance with previous publications [29, 32]. Black PS particles, which were stained slightly at the particle edges, were classified as MP suspect with a ß-error of 58 %. No particles from a milled red PP bottle cap were classified as MP suspect (ß-error 100 %). Inherent particle colour of polyester fibres influenced the observed fluorescence to a high extent. The fibres’ spectra often did not resemble the spectrum of the in-house PET reference even in approximation (e.g. green, blue, or violet fibres) as shown in Fig. S8. In contrast, the emission spectra of thin PP films were, albeit less bright compared to the PP reference, not influenced to a major extent by the inherent colour. Inherent colour of PNO particles can further result in a false-positive classification. For example, bright fluorescence was observed for yellow and orange cotton fibres. Orange cotton fibres showed even brighter fluorescence than orange PET fibres, as indicated in Fig. S9.

| 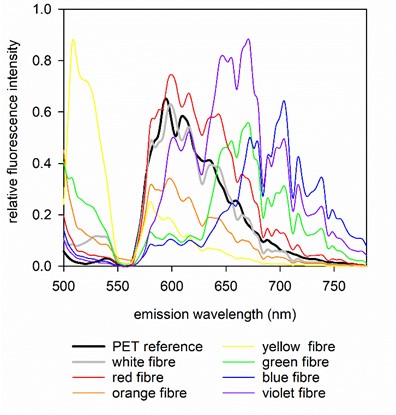 | 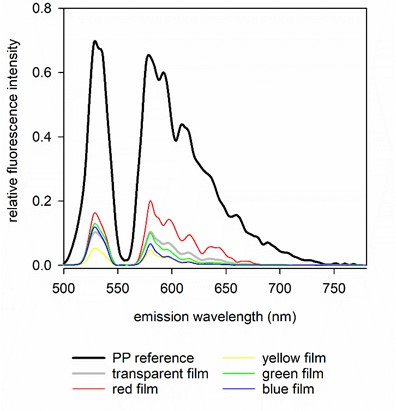 |
| --- | --- |

**Fig. S8** Emission spectra (λ_ex_ = 488 nm) of NR-stained polyester yarn (left) and PP-film (right) of different inherent colours.


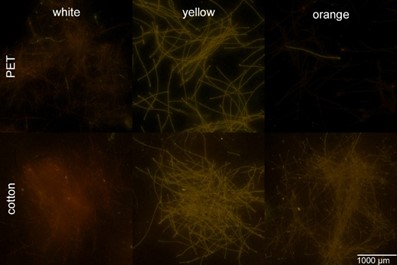


**Fig. S9** Photographs of Nile Red stained PET and cotton fibres of inherently white, yellow, or orange colour, observed with fluorescence microscopy.

### Impact of artificial UV-aging on polymer classification

Ageing of particles in the environment not only increases surface roughness, but also result in an increase of chemical polarity due to photooxidation [52], raising concerns regarding the viability of MP detection [43, 53]. Within the present study, artificial UV-aging of polymers led to a significant decrease of TPB for all tested polymers. While the proportion of red fluorescence increased significantly for PE(LD), PP, PS, and PVC, it decreased for PA6. A red-shift of NR fluorescence indicated an increase in the chemical polarity its surrounding [19], caused by photooxidation of the polymers [20]. The classification as MP suspect was not affected to a higher extent for PET, PS, and PVC with a ß-error of 0 %. In contrast, aging hampered MP suspect classification of PA6 and PE(LD) significantly with ß-errors of 98 % and 100 % respectively. An overview of fluorescence changes due to UV-aging is shown in Table S6.

**Table S6** Overview of significant influences (α = 0.05) on fluorescence properties (brightness, proportion of red fluorescence) after artificial UV-aging of synthetic polymers and ß-error of plastic identification

| **polymer** | **brightness** | | **red fluorescence** | | **plastic identification** | |
| --- | --- | --- | --- | --- | --- | --- |
|  | 26 h UV | 72 h UV | 26 h UV | 72 h UV | 26 h UV | 72 h UV |
| PA6 | decreased | decreased | - | decreased | ß = 98 % | ß = 100 % |
| LDPE | decreased | decreased | increased | increased | ß = 100 % | ß = 100 % |
| PET | decreased | decreased | - | - | ß = 0 % | ß = 0 % |
| PP | decreased | decreased | - | increased | ß = 31 % | ß = 16 % |
| PS | decreased | decreased | increased | - | ß = 0 % | ß = 0 % |
| PVC | decreased | decreased | decreased | increased | ß = 0 % | ß = 0 % |

## Optimisation of staining and measurement conditions for the application to seafood

### Assessment of dye concentration, incubation time, and temperature

Since the optimisation of NR-staining in general was subject of many previous studies [21-23], staining conditions for MP extracted from seafood matrices were only tested briefly. The most relevant polymers as well as chitin (PNO with greatest potential for interference) were stained with concentrations from 0.1 µg/mL to 1,000 µg/mL NR in isopropanol for 30 min at 40 °C [23, 24]. Furthermore, a temperature range between 20 °C and 70 °C and incubation times ranging from 10 min to 30 min were tested exemplarily [17, 21, 25].

Increased dye concentrations, incubation time, and temperature all resulted in higher particle brightness of pure polymers. Preliminary tests indicated that particle brightness did not increase significantly when staining with 1,000 µg/mL compared to 100 µg/mL NR in isopropanol, as illustrated in Fig. S11. However, high dye concentrations also increased background fluorescence, potentially masking weakly stained particles. In the range of 0.1 µg/mL to 100 µg/mL dye concentration and particle brightness of synthetic polymers as well as interfering PNO correlated linear (*R²*= 0.90–0.99). Therefore, a NR concentration of 100 µg/mL was chosen in the following. Comparing incubation times of 10 min and 30 min indicated higher fluorescence of some polymers (e.g. PVC). Consequently, samples were incubated for 30 min in the following, as all relevant polymers were stained to a sufficient degree and even longer incubation can result in interferences and increased background fluorescence [26]. A preliminary assessment of staining temperature impact at 20 °C and 40 °C indicated better staining at higher temperatures illustrated Fig. S12. This effect was most prominent for PVC, however, analysis of TPB in the range of 20 °C to 70 °C revealed no significant increase. Therefore, an incubation temperature of 40 °C was chosen in the following to avoid potential quenching of NR fluorescence by higher temperatures [27].


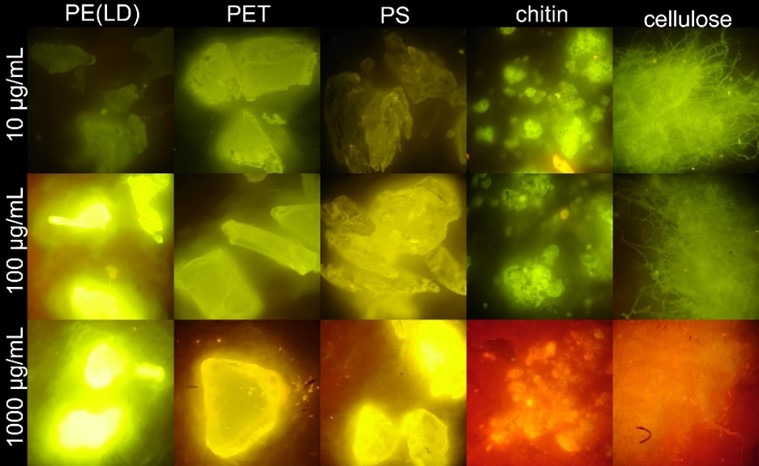


**Fig. S10** Photograph of particles stained with different concentrations of NR dissolved in isopropanol observed with FITC-filter cube and photographed with a smartphone camera via the ocular (iPhone SE 2020, no automatic exposure).


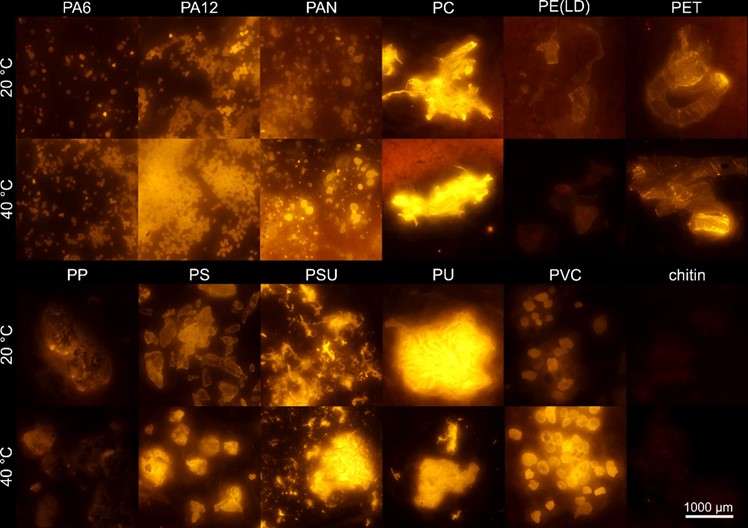


**Fig. S11** Photograph of particles stained with NR (1000 µg/mL in isopropanol) for 30 min at 20 °C and at 40 °C, observed with fluorescence microscopy (λ*_ex_* = 565 nm).

### Influence of solvent choice on fluorescence

Solvents of different polarity and molecular size were tested for staining. Due to the low solubility of NR in some solvents like n-hexane [28], a stock solution of 1,000 µg/mL NR in acetone was prepared. The stock solution was then diluted with DI water [22], ethanol: acetone (1:1, *v*/*v*, further referred to as EtAc) [21], isopropanol, hexane [28], CS_2_, CHCl_3_ [24], or DCM [28] with a ratio of 1:10. Plastic particles smaller than 500 µm from Goodfellow and AlfaAesar (Table S1, Nos. 1–12) as well as ground chitin were placed into a 96-well multiwell plate and suspended in 100 µL NR solution to ensure identical staining conditions. If necessary, the wells were mixed with a needle to disperse the particles thoroughly. After incubation for 30 min at 40 °C, excess solvent was removed using lint-free cellulose tissues and the samples were dried at room temperature. Subsequently, particles were transferred to clear, adhesive PP film and fixed onto black paper. The samples where then photographed in a UV-box (*λ*_ex_ ≈ 365 nm). Taking all tested synthetic polymer types into account, best staining results for differentiating PNO and plastics were achieved with NR dissolved in hexane, followed by EtAc, and CS_2_. In contrast, NR dissolved in isopropanol or water resulted in high TPB of chitin but low TPB of synthetic polymers. Solvent-dependent TBP for each polymer is illustrated in Fig. S13. All solvents except from water and isopropanol resulted in morphological deviations of certain polymers. Slight dissolution or swelling (change of particle shape) was observed when staining with EtAc (PC), and hexane (PS, especially expanded PS). Complete dissolution (formation of a polymer film after drying) was observed when staining with CS_2_ (PSU), as well as DCM and CHCl_3_ (PC, PSU, PU, PVC). Due to the different chemical polarity, best staining results of MP was achieved with a similar concentration of NR when staining with hexane- and EtAc-solution consecutively (Fig. S14).

| 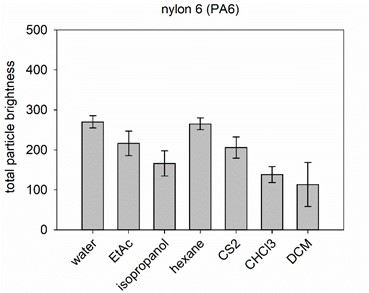 | 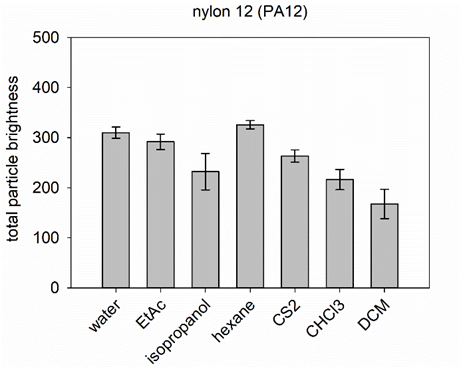 |
| --- | --- |
| 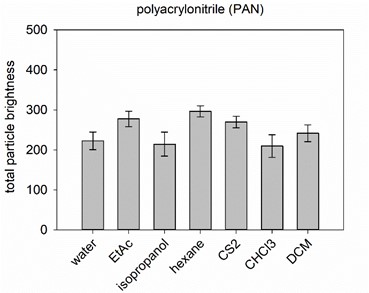 | 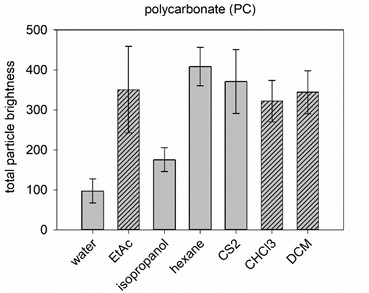 |
| 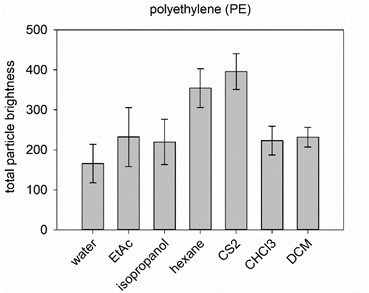 | 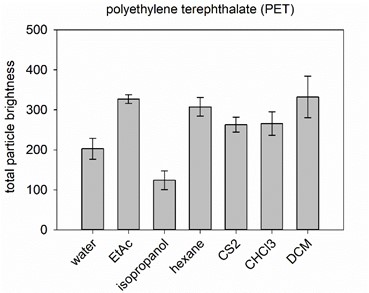 |
| 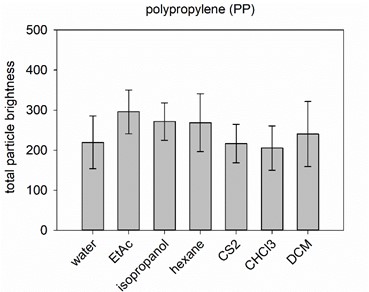 | 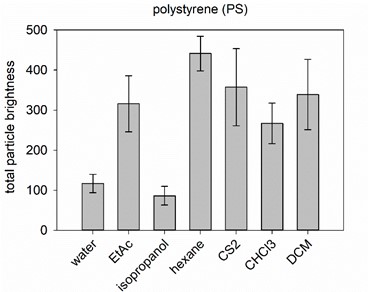 |
| 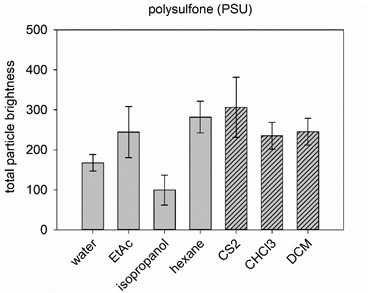 | 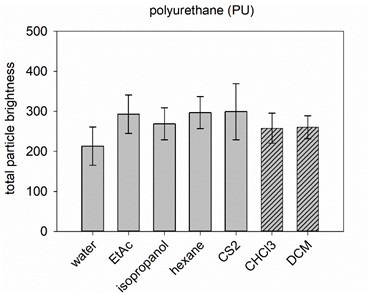 |
| 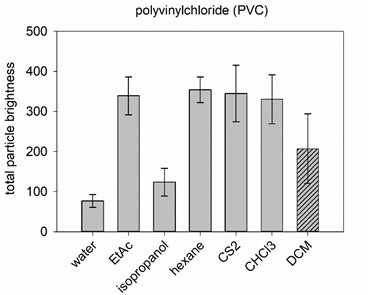 | 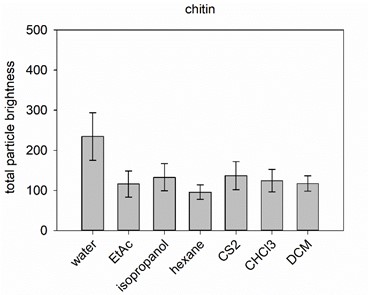 |

**Fig. S12** Total particle brightness of particles from synthetic and natural origin stained with 100 µg/mL NR in solvents of different polarity and molecular size (30 min incubation at 40 °C) and observed with a UV-box (λ_ex_ ≈ 365 nm). Hatched bars indicate observable swelling or dissolution of polymers.


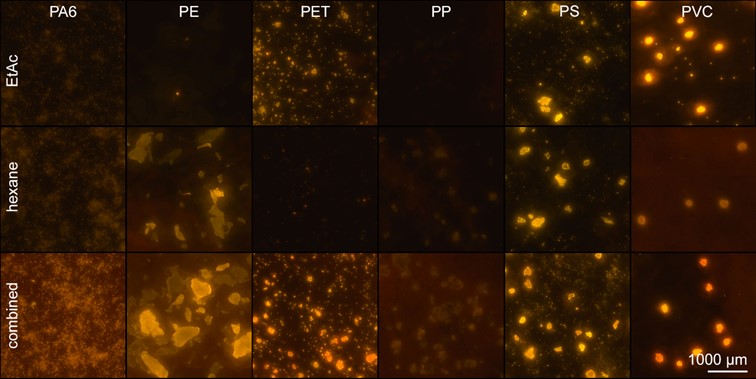


**Fig. S13** Unedited photographs of in-house reference MP stained two times with 100 µL of a 100 µg/mL NR-solution in EtAc and hexane each. For the combined approach, samples were stained once with 100 µL of each solution. Samples were observed with fluorescence microscopy.

### Impact of staining on polystyrene particles

As the PS used for evaluating the solvent choice for NR staining on fluorescence (section 2.5.2) was cross-linked (Table 1, No. 5), the tested particles were insoluble. However, the sensitivity of PS towards dissolution by organic solvents is widely known. Therefore, NR dissolved in different solvents was pipetted on household PS-MP (Table 1, No. 22, and No. 23) placed on a glass slide. The particles were then observed with light microscopy. Polymer dissolution was assessed visually as illustrated in Fig. S6 and indicated by a loss of (irregular) shape and surface structure. PS dissolution was especially prominent in contact with acetone and dichloromethane (DCM).


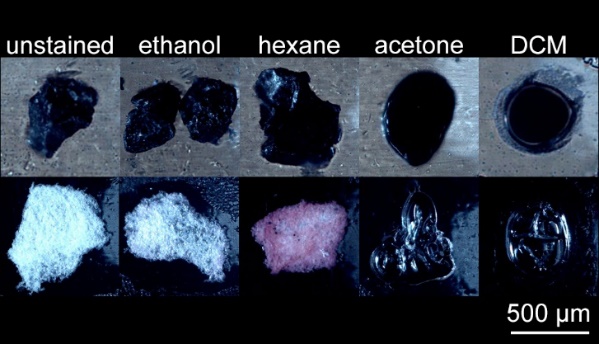


**Fig. S14** Light microscopic images of PS particles derived from black PS-packaging (upper row) and expanded polystyrene flakes (lower row) after staining with NR dissolved in different solvents.


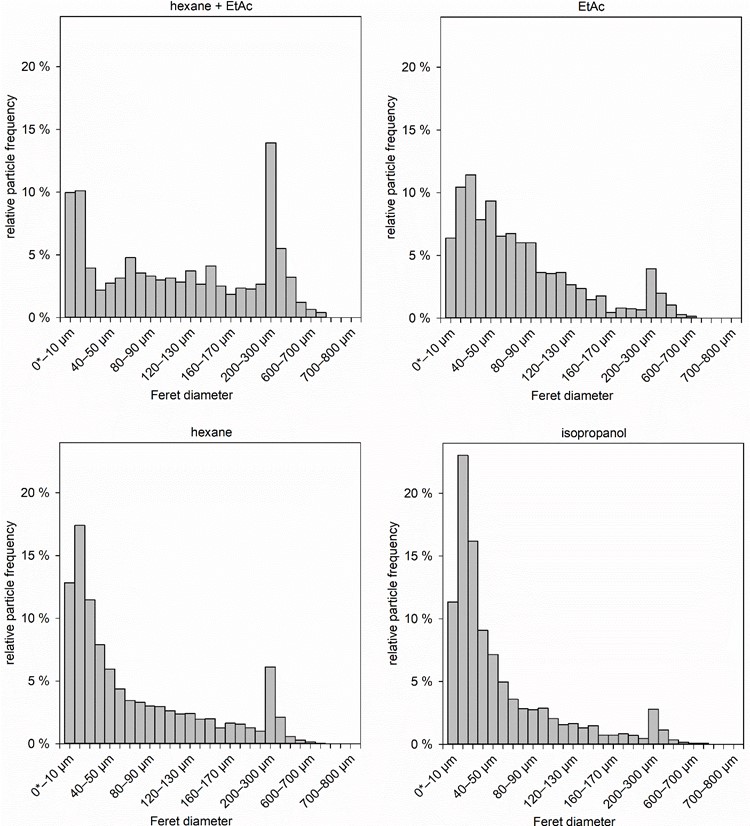


**Fig. S15** Particle size distribution of BAM-MP (n > 1000 particles) after staining with NR dissolved in isopropanol, hexane, ethanol:acetone (EtAc; 1:1, v/v) under the same conditions. *0–10 µm – despite 10 µm-intervals were set for binning, the realistic size range is 5–10 µm due to the microscope’s resolution.

### Evaluation of excitation wavelengths

For evaluating suitable excitation wavelengths, emission spectra of in-house reference materials and PNO were obtained with confocal scanning microscopy. Only green and cyan laser light (552 nm and 488 nm respectively) resulted in suitable fluorescence emission with a maximum in the range of 600 nm to 650 nm for most polymers as illustrated in Fig. S16. Polyolefins had an additional peak in the range of 500 nm to 550 nm. Excitation with green laser light (*λ*_ex_ = 552 nm) led to the brightest particle fluorescence for most polymers, but also PNO fluorescence was greatest. Excitation with cyan light turned out to be preferable (*λ*_ex_ = 488 nm) for detecting polyolefins (PE, PP), instead.

| 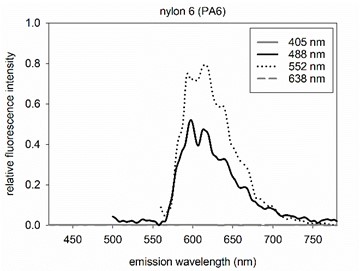 | 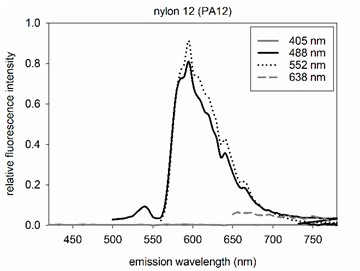 |
| --- | --- |
| 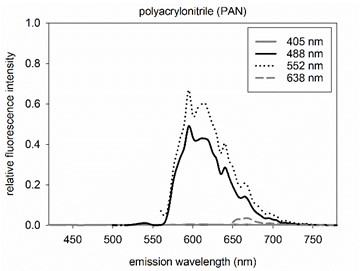 | 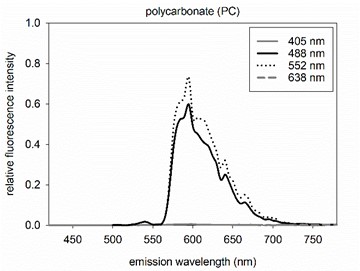 |
| 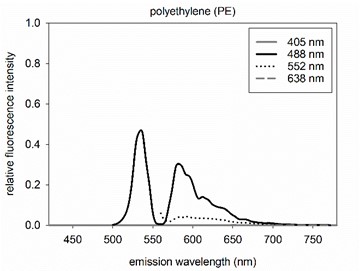 | 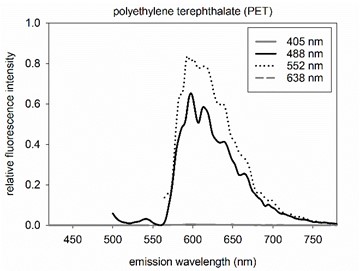 |
| 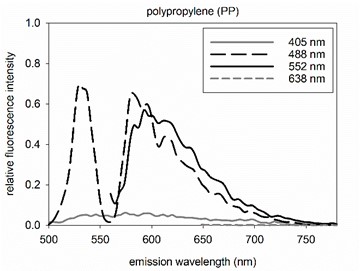 | 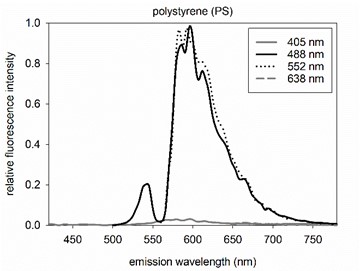 |
| 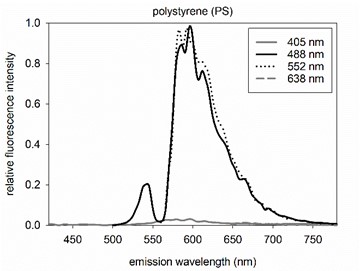 | 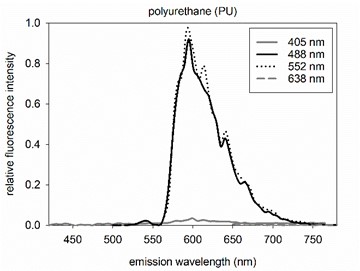 |
| 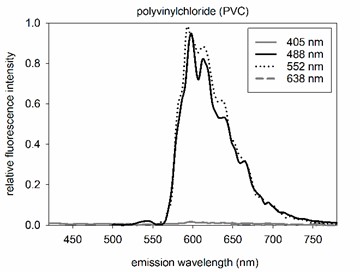 |  |
| 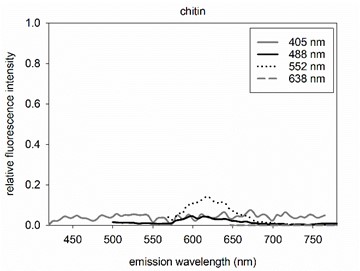 | 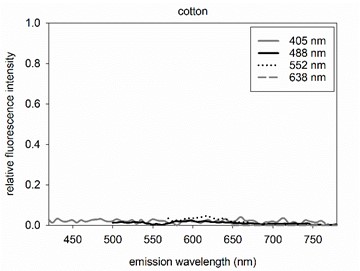 |
| 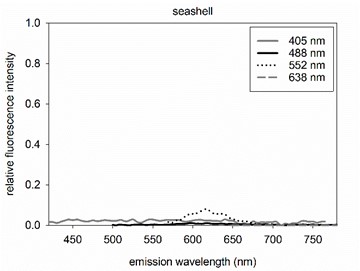 | 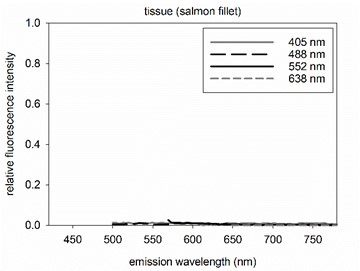 |

**Fig. S16** Emission spectra of commercially relevant polymers and particles of natural origin from possible procedural contaminants or matrix residues after Nile Red staining (1 mg/mL in isopropanol) observed with excitation wavelengths of 405 nm, 488 nm, 552 nm and 638 nm.

# Supplementary information on particle recovery

## Procedural contamination

**Table S8** Mean number of MP suspect particles in procedural blank samples procedural blank samples depending on size, fluorescence, and shape. Estimated particle mass is independent from particle shape or size. med. – medium

|  |  | **5–10 µm** | | | **10–50 µm** | | | **50–100 µm** | | | **100–500 µm** | | | **500–1000 µm** | | |
| --- | --- | --- | --- | --- | --- | --- | --- | --- | --- | --- | --- | --- | --- | --- | --- | --- |
| *fluorescence* | | *low* | *med.* | *high* | *low* | *med.* | *high* | *low* | *med.* | *high* | *low* | *med.* | *high* | *low* | *med.* | *high* |
| **series** | **shape** |  |  |  |  |  |  |  |  |  |  |  |  |  |  |  |
| 1 g-sample | spherical |  | 1 |  |  |  |  |  |  |  |  |  |  |  |  |  |
|  | rounded |  | 2 |  |  | 2 | 2 |  |  |  |  |  |  |  |  |  |
|  | angular |  |  |  |  | 1 |  |  | 1 |  |  |  |  |  |  |  |
|  |  | *low* | *med.* | *high* |  |  |  |  |  |  |  |  |  |  |  |  |
|  | mass estimate (µg) | 0.0 | 0.1 | 0.3 |  |  |  |  |  |  |  |  |  |  |  |  |
|  |  |  |  |  |  |  |  |  |  |  |  |  |  |  |  |  |
| PA12 | spherical | 5 | 2 |  |  |  |  |  |  |  |  |  |  |  |  |  |
|  | rounded |  | 13 | 3 |  | 14 | 1 |  | 11 | 1 |  | 2 |  |  |  |  |
|  | angular |  |  |  |  | 5 | 1 |  | 5 | 1 |  | 1 |  |  |  |  |
|  | irregular |  |  |  |  | 2 |  |  |  |  |  |  |  |  |  |  |
|  |  | *low* | *med.* | *high* |  |  |  |  |  |  |  |  |  |  |  |  |
|  | mass estimate (µg) | 0.0 | 5.4 | 0.2 |  |  |  |  |  |  |  |  |  |  |  |  |
|  |  |  |  |  |  |  |  |  |  |  |  |  |  |  |  |  |
| BAM-MP | spherical |  | 50 |  |  |  |  |  |  |  |  |  |  |  |  |  |
|  | rounded |  | 62 |  |  | 116 |  |  | 3 |  |  | 1 |  |  |  |  |
|  | angular |  |  |  |  | 64 |  |  | 11 |  |  | 4 |  |  |  |  |
|  | irregular |  |  |  |  | 27 |  |  | 7 |  |  | 2 |  |  |  |  |
|  | fibre (elongated) |  |  |  |  |  |  |  |  |  |  | 2 |  |  |  |  |
|  | fibre (coiled) |  |  |  |  |  |  |  |  |  |  | 1 |  |  |  |  |
|  |  | *low* | *med.* | *high* |  |  |  |  |  |  |  |  |  |  |  |  |
|  | mass estimate (µg) | 0.0 | 8.5 | 0.0 |  |  |  |  |  |  |  |  |  |  |  |  |
|  |  |  |  |  |  |  |  |  |  |  |  |  |  |  |  |  |
| MP mixture | spherical | 65 | 130 | 1 |  |  |  |  |  |  |  |  |  |  |  |  |
|  | rounded | 91 | 176 | 1 | 145 | 294 | 17 |  |  |  |  |  |  |  |  |  |
|  | angular |  |  |  | 26 | 150 | 4 | 2 | 31 | 1 | 1 | 17 |  |  |  |  |
|  | irregular |  |  |  | 3 | 72 |  |  | 25 | 1 |  | 6 |  |  |  |  |
|  | fibre (elongated) |  |  |  |  |  |  |  |  |  |  | 13 |  |  | 2 |  |
|  | fibre (coiled) |  |  |  |  |  |  |  |  |  |  | 4 |  |  | 2 |  |
|  |  | *low* | *med.* | *high* |  |  |  |  |  |  |  |  |  |  |  |  |
|  | mass estimate (µg) | 2.5 | 42 | 5.0 |  |  |  |  |  |  |  |  |  |  |  |  |
|  |  |  |  |  |  |  |  |  |  |  |  |  |  |  |  |  |

**Table S9** Quantification limits (LOQ) of MP suspect particles in procedural blank samples procedural blank samples depending on size, fluorescence, and shape. Estimated particle mass is independent from particle shape or size. med. – medium

|  |  | **5–10 µm** | | | **10–50 µm** | | | **50–100 µm** | | | **100–500 µm** | | | **500–1000 µm** | | |
| --- | --- | --- | --- | --- | --- | --- | --- | --- | --- | --- | --- | --- | --- | --- | --- | --- |
| *fluorescence* | | *low* | *med.* | *high* | *low* | *med.* | *high* | *low* | *med.* | *high* | *low* | *med.* | *high* | *low* | *med.* | *high* |
| **series** | **shape** |  |  |  |  |  |  |  |  |  |  |  |  |  |  |  |
| 1 g-sample | spherical | 6 | 6 |  |  |  |  |  |  |  |  |  |  |  |  |  |
|  | rounded | 6 | 2 |  | 18 | 25 |  |  |  |  |  |  |  |  |  |  |
|  | angular |  |  |  | 17 | 6 |  | 6 | 6 |  | 6 | 12 |  |  |  |  |
|  |  | *low* | *med.* | *high* |  |  |  |  |  |  |  |  |  |  |  |  |
|  | mass estimate (µg) | 2.0 | 5.2 |  |  |  |  |  |  |  |  |  |  |  |  |  |
|  |  |  |  |  |  |  |  |  |  |  |  |  |  |  |  |  |
| PA12 | spherical | 6 | 35 | 21 |  |  |  |  |  |  |  |  |  |  |  |  |
|  | rounded | 6 | 58 | 29 |  | 59 | 17 | 6 | 46 | 11 | 27 | 6 |  |  |  |  |
|  | angular |  |  |  |  | 35 | 6 |  | 10 | 6 |  | 17 | 6 |  |  |  |
|  | irregular |  |  |  |  | 13 |  |  | 6 |  |  |  |  |  |  |  |
|  |  | *low* | *med.* | *high* |  |  |  |  |  |  |  |  |  |  |  |  |
|  | mass estimate (µg) | 0.3 | 60 | 3.0 |  |  |  |  |  |  |  | 6 |  |  |  |  |
|  |  |  |  |  |  |  |  |  |  |  |  |  |  |  |  |  |
| BAM-MP | spherical |  | 706 |  |  |  |  |  |  |  |  |  |  |  |  |  |
|  | rounded |  | 909 |  |  | 1507 |  |  | 32 |  |  | 13 |  |  |  |  |
|  | angular |  |  |  |  | 861 |  |  | 103 |  |  | 36 |  |  |  |  |
|  | irregular |  |  |  |  | 356 |  |  | 96 |  |  | 43 |  |  |  |  |
|  | fibre (elongated) |  |  |  |  |  |  |  |  |  |  | 12 |  |  | 6 |  |
|  | fibre (coiled) |  |  |  |  |  |  |  |  |  |  | 12 |  |  |  |  |
|  |  | *low* | *med.* | *high* |  |  |  |  |  |  |  |  |  |  |  |  |
|  | mass estimate (µg) | 0.0 | 118 | 0.0 |  |  |  |  |  |  |  |  |  |  |  |  |
|  |  |  |  |  |  |  |  |  |  |  |  |  |  |  |  |  |
| MP mixture | spherical | 1034 | 683 | 17 |  | 6 |  |  |  |  |  |  |  |  |  |  |
|  | rounded | 1476 | 581 | 18 | 2146 | 755 | 191 | 6 | 6 | 6 |  |  |  |  |  |  |
|  | angular |  |  |  | 309 | 961 | 24 | 22 | 626 | 6 | 7 | 215 | 6 |  |  |  |
|  | irregular |  |  |  | 53 | 754 |  | 6 | 320 | 17 |  | 99 |  |  |  |  |
|  | fibre (elongated) |  |  |  |  |  |  |  |  |  |  | 123 |  |  | 34 |  |
|  | fibre (coiled) |  |  |  |  |  |  |  |  |  |  | 65 |  |  | 19 |  |
|  |  | *low* | *med.* | *high* |  |  |  |  |  |  |  |  |  |  |  |  |
|  | mass estimate (µg) | 35 | 694 | 87 |  |  |  |  |  |  |  |  |  |  |  |  |
|  |  |  |  |  |  |  |  |  |  |  |  |  |  |  |  |  |

## Comparison of fluorescence imaging and LDIR imaging particle counting

Most particles were identified as PET (in mean 69 %), followed by PVC (17 %), and PE (7 %). The proportion of PS particles varied between samples with 11 % and 2 % respectively. Only one PP particle was detected in sample 2. In addition to the spiked polymer types, one polycarbonate particle was detected in each sample. Particles identified as PA were not counted as MP, as LDIR was not able to differentiate them from natural peptides present in the investigated seafood samples. Furthermore, several particles were identified as “rubber”. As fatty acids interfere with the identification of rubber particles, they were not counted as MP as well.

**Table S10** Particle numbers of sizes and polymer types according to fluorescence imaging and LDIR imaging. Identification of polymer types was not feasible with fluorescence imaging. *not counted as differentiation from PNO was impossible

|  | **sample 1** | | **sample 2** | |
| --- | --- | --- | --- | --- |
|  | fluorescence | LDIR | fluorescence | LDIR |
| total particle number | 92 | 10820 | 610 | 14692 |
| MP suspect number | 23 | 72 | 134 | 161 |
|  |  |  |  |  |
| 10–50 µm | 17 | 21 | 111 | 26 |
| 50–100 µm | 0 | 25 | 9 | 45 |
| 100–200 µm | 0 | 19 | 9 | 72 |
| 200–500 µm | 6 | 7 | 5 | 17 |
| > 500 µm | 0 | 0 | 0 | 1 |
|  |  |  |  |  |
| PA6 / proteins | - | 40* | - | 86* |
| PE | - | 4 | - | 12 |
| PET | - | 48 | - | 113 |
| PP | - | 0 | - | 1 |
| PS | - | 8 | - | 4 |
| PVC | - | 11 | - | 30 |
| PC | - | 1 | - | 1 |
| rubber / fatty acids | - | 10 | - | 48 |

# References

1. PlasticsEurope. Plastics - The Facts 2021. An analysis of European plastics production, demand and waste data2021.

2. Kühn S, van Oyen A, Booth AM, Meijboom A, van Franeker JA. Marine microplastic: Preparation of relevant test materials for laboratory assessment of ecosystem impacts. Chemosphere. 2018;213:103-13.

3. Balakrishnan G, Déniel M, Nicolai T, Chassenieux C, Lagarde F. Towards more realistic reference microplastics and nanoplastics: preparation of polyethylene micro/nanoparticles with a biosurfactant. Environmental Science: Nano. 2019;6(1):315-24.

4. Ziani K, Ioniță-Mîndrican C-B, Mititelu M, Neacșu SM, Negrei C, Moroșan E, et al. Microplastics: A Real Global Threat for Environment and Food Safety: A State of the Art Review. Nutrients [Internet]. 2023; 15(3).

5. Erni-Cassola G, Gibson MI, Thompson RC, Christie-Oleza JA. Lost, but Found with Nile Red: A Novel Method for Detecting and Quantifying Small Microplastics (1 mm to 20 μm) in Environmental Samples. Environmental Science & Technology. 2017;51(23):13641-8.

6. Süssmann J, Krause T, Martin D, Walz E, Greiner R, Rohn S, et al. Evaluation and optimisation of sample preparation protocols suitable for the analysis of plastic particles present in seafood. Food Control. 2021;125:107969.

7. Yu JT, Diamond ML, Helm PA. A fit-for-purpose categorization scheme for microplastic morphologies. Integrated Environmental Assessment and Management. 2023;19(2):422-35.

8. Mingqiang Y, Kidiyo K, Joseph R. A survey of shape feature extraction techniques. Pattern recognition. 2008;15(7):43-90.

9. Wirth M. Shape Analysis & Measurement Shape Analysis & Measurement. Image Processing. 2004:1-49.

10. Villarino MB. A note on the accuracy of Ramanujan’s approximative formula for the perimeter of an ellipse. Journal of Inequalities in Pure and Applied Mathematics. 2006;7(1(21)):1-10.

11. Takashimizu Y, Iiyoshi M. New parameter of roundness R: circularity corrected by aspect ratio. Progress in Earth and Planetary Science. 2016;3(1):2.

12. Jarvis RA. On the identification of the convex hull of a finite set of points in the plane. Information Processing Letters. 1973;2(1):18-21.

13. Ismayilova I, Zeyer T, Timpf S. Identification of Microplastics in Soils Using 2D Geometric Shape Descriptors. AGILE: GIScience Series. 2021;2:1-6.

14. Chen S, Li Y, Mawhorter C, Legoski S. Quantification of microplastics by count, size and morphology in beverage containers using Nile Red and ImageJ. Journal of Water and Health. 2021;19(1):79-88.

15. Braun U. Analysis of Microplastics: Sampling, preparation and detection. Berlin, Germany; 2021 May 2021.

16. Nel HA, Chetwynd AJ, Kelleher L, Lynch I, Mansfield I, Margenat H, et al. Detection limits are central to improve reporting standards when using Nile red for microplastic quantification. Chemosphere. 2021;263:127953.

17. Maes T, Jessop R, Wellner N, Haupt K, Mayes AG. A rapid-screening approach to detect and quantify microplastics based on fluorescent tagging with Nile Red. Scientific Reports. 2017;7:44501.

18. Toppe J, Albrektsen S, Hope B, Aksnes A. Chemical composition, mineral content and amino acid and lipid profiles in bones from various fish species. Comparative Biochemistry and Physiology Part B: Biochemistry and Molecular Biology. 2007;146(3):395-401.

19. Sackett DL, Wolff J. Nile red as a polarity-sensitive fluorescent probe of hydrophobic protein surfaces. Analytical biochemistry. 1987;167(2):228-34.

20. Gewert B, Plassmann MM, MacLeod M. Pathways for degradation of plastic polymers floating in the marine environment. Environmental Science: Processes & Impacts. 2015;17(9):1513-21.

21. Konde S, Ornik J, Prume JA, Taiber J, Koch M. Exploring the potential of photoluminescence spectroscopy in combination with Nile Red staining for microplastic detection. Mar Pollut Bull. 2020;159:111475.

22. Gao Z, Wontor K, Cizdziel JV. Labeling Microplastics with Fluorescent Dyes for Detection, Recovery, and Degradation Experiments. Molecules. 2022;27(21):7415.

23. Hernandez LM, Farner JM, Claveau-Mallet D, Okshevsky M, Jahandideh H, Matthews S, et al. Optimizing the Concentration of Nile Red for Screening of Microplastics in Drinking Water. ACS ES&T Water. 2023;3(4):1029-38.

24. Tamminga M, Hengstmann E, Fischer EK. Nile Red Staining as a Subsidiary Method for Microplastic Quantification: A Comparison of Three Solvents and Factors Influencing Application Reliability - short communication. SDRP Journal of Earth Sciences & Environmental Studies. 2017;2(2):8.

25. Gao Z, Cizdziel JV, Chen L. Microplastics profile in sludge from a university wastewater treatment plant and the influence of chemical digestions on Nile red stained microplastics. Journal of Environmental Chemical Engineering. 2023;11(3):109671.

26. Shruti VC, Pérez-Guevara F, Roy PD, Kutralam-Muniasamy G. Analyzing microplastics with Nile Red: Emerging trends, challenges, and prospects. Journal of hazardous materials. 2022;423:127171.

27. Alemán-Nava GS, Cuellar-Bermudez SP, Cuaresma M, Bosma R, Muylaert K, Ritmann BE, Parra R. How to use Nile Red, a selective fluorescent stain for microalgal neutral lipids. Journal of microbiological methods. 2016;128:74-9.

28. Shim WJ, Song YK, Hong SH, Jang M. Identification and quantification of microplastics using Nile Red staining. Marine Pollution Bulletin. 2016;113(1):469-76.
